# AKR1B1 Expression in the Colorectal Tumor Microenvironment Contributes Towards Its Prognostic Significance

**DOI:** 10.1002/cam4.70974

**Published:** 2025-05-21

**Authors:** Seçil Demirkol Canlı, Güneş Güner, Aynur Işık, Branka Sosic‐Jurjevi, Aleksandra Djikic Rom, Esin Gülce Seza, Ömer Dizdar, Sandra Dragicevic, Aleksandra Nikolic, Aytekin Akyol, Sreeparna Banerjee

**Affiliations:** ^1^ Division of Tumor Pathology, Department of Clinical Oncology Cancer Institute, Hacettepe University Ankara Turkiye; ^2^ Department of Pathology Hacettepe University Faculty of Medicine Ankara Turkiye; ^3^ Transgenic Animal Technologies Research and Application Center Hacettepe University Ankara Turkiye; ^4^ Department of Cytology Institute for Biological Research “Siniša Stanković”‐National Institute of Republic of Serbia, University of Belgrade Belgrade Serbia; ^5^ Department of Pathology, Pathohistology and Medical Cytology University Clinical Center of Serbia Belgrade Serbia; ^6^ Department of Biological Sciences Orta Dogu Teknik Universitesi Ankara Turkiye; ^7^ Department of Medical Oncology Hacettepe University Cancer Institute Ankara Turkiye; ^8^ Gene Regulation in Cancer Group Institute of Molecular Genetics and Genetic Engineering, University of Belgrade Belgrade Serbia

**Keywords:** AKR1B1, colorectal cancer, gene expression, prognosis, stroma, tissue microenvironment

## Abstract

**Background:**

AKR1B1, a member of the aldo‐keto reductase enzyme family involved in the polyol pathway of aldehyde metabolism, is aberrantly expressed in colorectal cancer (CRC). Our previous studies demonstrated that AKR1B1 knockdown reduced the motility and proliferation of CRC cell lines, and its elevated expression was correlated with increased mesenchymal marker expression, inflammation, and poor prognosis in CRC patient cohorts. However, whether stromal cells also express AKR1B1 and whether stromal expression can affect clinical outcomes has not been examined.

**Objectives:**

To evaluate the expression of AKR1B1 within the tumor microenvironment (TME) of CRC, with a paticular focus on stromal cells, and to assess its association with clinical outcomes.

**Methods:**

We assessed AKR1B1 expression in colorectal tumors utilizing publicly available transcriptomic data from CRC tumors. Single‐cell RNA‐sequencing data from CRC samples were analyzed to determine cell type‐specific expression. Immunohistochemistry based assessment of AKR1B1 expression was performed in Turkish and Serbian cohorts. Co‐localization of AKR1B1 and CD163 (M2 macrophage marker) was evaluated by immunoflourescence.

**Results:**

AKR1B1 was expressed in both the epithelial and stromal components of colorectal tumors, with higher expression observed in the stroma. Single‐cell transcriptomic analysis revealed AKR1B1 expression in myeloid cells, T and NK cells, B cells, dendritic cells, fibroblasts, and epithelial cells. Notably, AKR1B1‐expressing macrophages were predominantly of the M2 phenotype, and AKR1B1 expression and M2 marker expression showed strong positive correlation in bulk transcriptomic data. Immunofluorescence confirmed the colocalization of CD163 and AKR1B1 in stromal macrophages. Moreover, immunohistochemical analysis of AKR1B1 expression in tumor stroma from a cohort of Turkish patients revealed that its expression was associated with favorable overall survival, particularly in tumors with higher stromal infiltration.

**Conclusions:**

Overall, our findings underscore the significant influence of the TME composition on the relationship between AKR1B1 expression and clinical outcomes.

## Graphical abstract

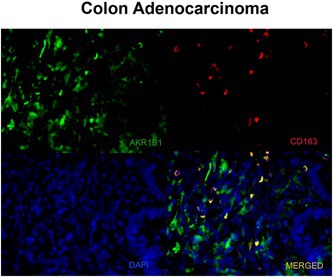

## 1 Introduction

Colorectal cancer (CRC) is one of the most prevalent malignancies worldwide. Understanding the molecular mechanisms in the tumor microenvironment (TME) may help in the development of more effective therapies. CRC is distinct from most tumors due to its interplay with the gut microbiota, which plays a crucial role in shaping the TME. In addition to the microbiome, CRC cells also interact with various cellular components such as immune cells, cancer‐associated fibroblasts, and endothelial cells in either promoting or suppressing tumor development [1].

Aldo‐keto reductases (AKRs) are a superfamily of enzymes that catalyze an extensive range of oxidation–reduction reactions. They are mainly cytosolic, monomeric (34–37 kDa), nicotinamide adenine dinucleotide phosphate (NADPH)‐dependent oxidoreductases and are involved in many critical metabolic events in health and disease [2]. AKRs can reduce aldehydes and ketones (carbonyl groups), including glucose, retinals, and a number of drugs to their corresponding primary and secondary alcohols [3].

Previously, we have shown that high AKR1B1 mRNA expression in bulk colorectal tumor specimens was associated with worse prognosis, which could be validated using publicly available transcriptome data from multiple datasets [4]. Mechanistically, we showed that AKR1B1 expression was associated with high mitogenic signaling via the MAP Kinase pathway as well as rapid cell cycle progression, along with the activation of NF‐κB and inflammation [4, 5]. Other studies have shown that AKR1B1 may play a key role in the progression of colitis to CRC, further highlighting a role of AKR1B1 in inflammation [6]. High expression of AKR1B1 was also associated with worse prognosis in gastric cancer [7] and breast cancer [8], and with favorable prognosis in endometrial cancer (when combined with the expression of AKR1B10) [9].

However, relatively little is known about the expression of AKR1B1 in the CRC TME. One recent study has shown that high expression of AKR1B1 in gastric cancer cells was associated with immunosuppressive immune cell infiltration and worse prognosis, although it was not reported whether the stromal cells expressed any AKR1B1 [10]. Another study has reported the expression of Aldose Reductase (AR) in murine splenic T cells; AR was regulated via phosphorylation/dephosphorylation in order to transmit immunosuppressive signals to T cells from macrophages generated upon infection with the *Mycobacterium avium* complex [11]. In the current study, we aimed to understand whether AKR1B1 is expressed solely by cancer cells or also by other cell types in the TME, and whether its expression from the TME contributes to the prediction of prognosis.

## 2 Materials and Methods

An overview of the methods is presented here. Detailed information can be found in Appendix S4.

### 2.1 Patient Characteristics

The Turkish study cohort included formalin‐fixed paraffin‐embedded (FFPE) tumor tissues from 269 stage II or III CRC patients. The Serbian cohort consisted of 87 FFPE tumor tissues from CRC patients with early‐onset disease taken at surgery and 24 pairs of tumor and healthy mucosa samples from patients with locally advanced rectal cancer (LARC) taken at biopsy before administration of neoadjuvant therapy. Human tissue samples and clinical data from Turkish patients were deidentified and used retrospectively; informed consent was not required by the relevant ethics committee. Informed consent was obtained from all Serbian patients included in the study.

### 2.2 Immunohistochemistry

Immunohistochemistry of the Turkish cohort was carried out using citrate for the antigen retrieval procedure as described previously [12]. Fifteen tissue microarrays (TMA) that represented 3‐mm diameters of 2 cores from FFPE tissues of 264 primary CRC patients were used. The sections were processed and incubated with the AKR1B1 antibody (1:250; Thermo Fisher Scientific, PA5‐29718) overnight at 4°C.

### 2.3 Immunofluorescence Staining

FFPE tissues of normal colon and colon adenocarcinomas were sectioned into 4‐μm‐thick sections. The sections were processed and incubated separately with the AKR1B1 (Thermo Fisher Scientific, PA529718, 1:3200 dilution) and the CD163 (Ab156769, Abcam, Cambridge, MA, 1:800 dilution) antibodies overnight at 4°C, followed by visualization.

### 2.4 Cell Culture and Macrophage Differentiation

THP‐1 human monocytes were cultured as per ATCC recommendations. THP‐1 cells were differentiated into M0 macrophages using phorbol 12‐myristate 13‐acetate (PMA; Sigma‐Aldrich, St Louis, MO, USA) and then further differentiated into M2 macrophages using standard protocols.

### 2.5 Western Blot

Protein isolation and western blot were carried out using standard techniques. The primary antibodies AKR1B1 (Invitrogen; PA5‐29718), CD163 (Abcam; ab156769), and α‐Tubulin (Cell Signalling Technology; 9099S) were used at 1:1000, 1:1000, and 1:5000 concentrations, respectively, and visualized. Semi‐quantitative analysis of Western blot data was conducted using Image Lab Software (Bio‐Rad). Densitometric values were normalized to a loading control (α‐tubulin). Subsequent statistical analyses were performed based on the normalized data.

### 2.6 RNA Isolation and Real‐Time PCR

Total RNA was extracted from tumor tissues collected before neoadjuvant therapy at the First Surgical Clinic, Clinical Center of Serbia, Belgrade, Serbia using Trizol reagent (Ambion, Foster City, CA, USA). Fold changes in the expression of CDH1, VIM, and AKR1B1 were calculated with respect to the geometric mean of ACTB and GAPDH. Total RNA isolation from M2 differentiated macrophages was carried out as per standard protocols. The expression of Transglutaminase 2 (TGM2) was determined by quantitative real‐time PCR (qRT‐PCR). Primers are listed in Table S1.

### 2.7 Analysis of Gene Expression Data

Raw data for GSE17536, GSE39582, GSE39396, and GSE16385 datasets were downloaded from the NCBI GEO database (https://www.ncbi.nlm.nih.gov/geo/) and RMA normalized using “affy” package. To score CRC tumors for the level of immune and stromal cells in the TME, CIBERSORT and ESTIMATE algorithms were applied. Consensus molecular subtypes (CMS) information for GSE39582 was obtained from www.synapse.org.

### 2.8 Analysis of Prognostic Relationships

Log rank multiple cutoff (LRMC) graphs were generated as described previously [13]. Red and blue colors indicate the association of high expression with poor and good prognosis, respectively. Vertical dashed lines represent the 25 percentile, median, and 75th percentile values, whereas the horizontal dashed line denotes *p* < 0.05.

### 2.9 Evaluation of Single Cell RNA‐Sequencing (scRNA‐seq) Data From Colorectal Tumors

scRNA‐seq data were downloaded from GSE178318. Tumors of the three patients who were treatment‐naive were included in the analysis. The filtered data of GSE178341 were downloaded and used for visualization [14]. scRNA‐seq data from GSE146771 was analyzed with the web portal http://crcleukocyte.cancer‐pku.cn/ [15].

### 2.10 Statistical Analysis

Detailed descriptions of the statistical analyses are provided in Appendix S1.

## 3 Results

### 3.1 Expression of AKR1B1 by Stromal and Immune Cells in the Colorectal Tumor Microenvironment

**FIGURE 1:**
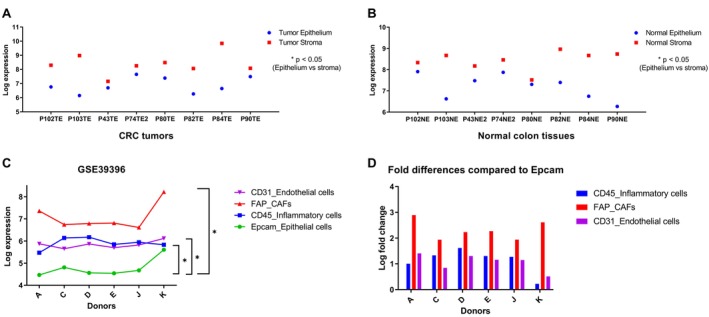
AKR1B1 expression in the CRC tumor microenvironment. AKR1B1 expression was determined in laser capture microdissected stromal and epithelial sections of CRC tumors (A) and paired adjacent normal colon tissues (B) (GSE31279). Sample identifiers combined patient numbers and sample types, such as “Patient 102 Tumor Expression” (P102TE) and “Patient 102 Normal Expression” (P102NE). AKR1B1 expression was significantly higher in the stromal compartment compared to epithelial compartment for both tumor and normal (Wilcoxon mached pairs signed rank test *p* value: 0.0078 for both comparisons). Normalized expression of AKR1B1 in FACS sorted cells of CRC tumors (GSE39396) (C). Log fold change of AKR1B1 expression in CAFs and inflammatory cells compared to epithelial cells (D). Donors are labeled with the letters A, C, D, E, J, and K. AKR1B1 expression was the lowest in epithelial cells in all samples (C), and significantly higher in the CAFs (median Log Fold Change [LFC] 2.25), inflammatory cells (LFC 1.29) and endothelial cells (LFC 1.15) (D) compared to the epithelial cells (*p < 0.05).

GSE31279 dataset was used to evaluate the expression of AKR1B1 in the CRC TME, which included eight laser microdissected tumors and paired normal colon tissues obtained from CRC patients. AKR1B1 expression was significantly higher in the stroma compared to the epithelium in all samples in CRC tumors as well as normal colon (*p* < 0.05) (Figure 1A,B). We next analyzed an independent microarray dataset (GSE39396) generated from cells obtained from the freshly dissected CRC tumors that were FACS sorted with cell‐specific markers (EPCAM for epithelial cells, CD45 for inflammatory cells, FAP for CAFs (cancer‐associated fibroblasts), and CD31 for endothelial cells). Indeed, AKR1B1 expression was the lowest in epithelial cells in all samples (Figure 1C) and significantly higher in the CAFs (median Log Fold Change [LFC] 2.25), inflammatory cells (LFC 1.29), and endothelial cells (LFC 1.15) (Figure 1D) compared to the epithelial cells. These data suggest that in CRC, AKR1B1 is expressed by CD45^+^ immune cells, FAP expressing CAFs, CD31^+^ endothelial cells, and to a lower extent, in epithelial cells. When AKR1B1 expression was quantified in paired rectal tumor and adjacent normal tissues obtained from Serbian patients, its expression was significantly higher in tumors (*p*: 0.002), suggesting higher expression of AKR1B1 in tumor‐associated stroma and/or transformed epithelial cells (Figure S1A).

**FIGURE 2:**
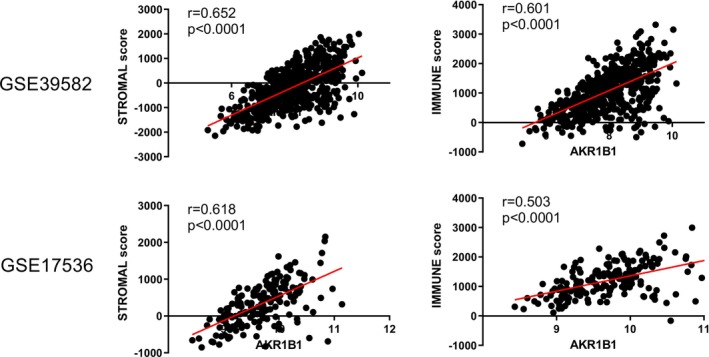
Correlation of AKR1B1 expression with stromal and immune scores. Scores generated via the ESTIMATE method are indicated in the *y*‐axes. AKR1B1 expression is shown in the *x*‐axes. Data from GSE39582 (upper panel) and GSE17536 (lower panel) are shown. Pearson *r* and *p* values are given for each correlation.

An analysis of immune and stromal scores generated by the in silico scoring method ESTIMATE confirmed these findings, revealing strong positive correlations of both scores with AKR1B1 expression (*r* > 0.50, *p* < 0.0001), suggesting that tumors with high immune and stromal content have higher AKR1B1 expression (Figure 2). In order to examine whether AKR1B1 expression from the neoplastic cells or tumor stroma is correlated specifically with stromal content, AKR1B1 expression was evaluated individually for neoplastic cells and tumor stroma via IHC in colon tumors obtained from Serbian patients. The data showed a significant positive correlation between stromal ratio and stromal AKR1B1 expression (Spearman rho: 0.226, *p*: 0.041), whereas expression from the epithelial cells revealed no significant relationship (*p* > 0.05). Evaluation of AKR1B1 expression along with previously defined consensus molecular subtypes (CMS) of CRC [16] showed that AKR1B1 expression was the highest in CMS4 type, followed by CMS1, CMS2, and CMS3 (Figure S1B, *p* < 0.01 for all comparisons) type tumors. Altogether, these results support that AKR1B1 expression is higher in tumor subgroups with a denser stromal and immune cell content, and it is expressed primarily in the non‐epithelial cells in the CRC TME.

### 3.2 Expression of AKR1B1 in the Myeloid Lineage

**FIGURE 3:**
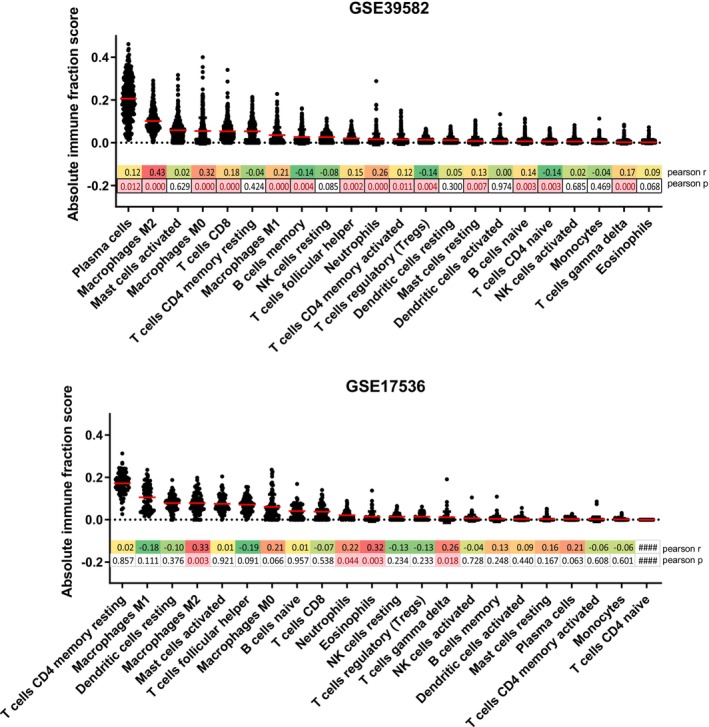
CIBERSORT‐based scores for 22 immune cell types in GSE39582 (upper panel) and GSE17536 (lower panel). 427 and 81 samples with deconvolution *p* value below 0.05 were included for GSE39582 and GSE17536, respectively. Pearson *p* and *r* values are given for correlation of each score with AKR1B1 expression. *p* values below 0.05 are shown in red. Pearson *r* values are colored in red, yellow, and green from highest value to the lowest. The red horizontal line reflects the mean score. Correlation *r* and *p* values could not be generated for the cells marked as ####.

We next aimed to identify which type of cell in the TME showed the highest expression of AKR1B1. For this, we utilized the CIBERSORT algorithm to estimate abundances of immune cell types based on gene expression data. The cell types with the highest score were plasma cells and CD4 memory T cells in the datasets GSE39582 and GSE17536, respectively (Figure 3). We next analyzed the linear correlation of these scores with AKR1B1 expression. Strikingly, M2 macrophages had the most significant positive correlation with AKR1B1 expression in both datasets (*r*: 0.43 and *r*: 0.33 for GSE39582 and GSE17536, respectively), indicating that tumors with a higher content of M2 macrophages in the TME showed an overall higher expression of AKR1B1 (Figure 3, Figure S2B–D). We also noted a significant positive correlation between M1 macrophage fractions and the expression of AKR1B1 in the dataset GSE39582 (*r*: 0.21, *p* < 0.0001) (Figure S2A); however, this relationship did not reach significance in GSE17536 (Figure S2C). These data suggest either that M2 macrophages can directly express AKR1B1, or that AKR1B1 expression can be induced in a TME that is rich in M2 macrophages. To clarify this, we utilized another microarray dataset, GSE16385, where transcriptome data from naïve macrophages differentiated into M1 and M2 types was available. Here, AKR1B1 expression was increased upon IL‐4 induction (M2 differentiation) and decreased upon IFNγ + TNFα induction (M1 differentiation) (Figure S2E). An analysis of the expression data of M1 and M2 macrophages differentiated from PBMCs obtained from healthy donors in GSE117040 indicated that both M1 and M2 macrophages expressed AKR1B1; however, the expression of AKR1B1 was higher in M2 macrophages in all donors compared to M1 macrophages (Figure S2F). Linear correlation analyses between four well‐known markers of M2 macrophages (CD206, CD204, CD163, ARG1) with AKR1B1 showed that 3 out of 4 markers were significantly and positively correlated with AKR1B1 expression in both GSE39582 and GSE17536 (*r* > 0.45, *p* < 0.001, Table 1), further supporting a strong relationship between the M2 macrophage population and AKR1B1 expression. Overall, our data suggest that AKR1B1 is expressed in myeloid tissues, primarily in M2 macrophages.

**TABLE 1:** Correlation of M2 macrophage markers with AKR1B1 expression.

Gene symbol	Probeset ID	GSE39582		GSE17536	
		*r*	*p*	*r*	*p*
MRC1 (CD206)	204438_at	0.539	< 0.0001	0.470	< 0.0001
MSR1 (CD204)	214770_at	0.477	< 0.0001	0.605	< 0.0001
MSR1 (CD204)	211887_x_at	0.474	< 0.0001	0.505	< 0.0001
MSR1 (CD204)	208423_s_at	0.420	< 0.0001	0.459	< 0.0001
MSR1 (CD204)	208422_at	0.139	0.001	0.002	0.970
CD163	203645_s_at	0.594	< 0.0001	0.560	< 0.0001
CD163	215049_x_at	0.582	< 0.0001	0.568	< 0.0001
CD163	216233_at	0.258	< 0.0001	0.212	0.005
ARG1	231665_at	0.017	0.683	0.021	0.777
ARG1	206177_s_at	0.003	0.936	−0.138	0.067
ARG1	231663_s_at	−0.056	0.182	−0.044	0.565
ARG1	231662_at	−0.075	0.076	−0.030	0.696

We next wanted to determine whether in vitro differentiated M2 macrophages could also show high AKR1B1 expression compared to the undifferentiated macrophages. For this, we differentiated THP‐1 monocytes to M2 macrophages via treatment with IL4 and IL13. We observed an increase in the expression of the M2 marker TGM2 by qRT‐PCR and the protein expression of CD163 by western blot (Figure S3A,C,D) [17, 18]. We did not note a significant change in the expression of AKR1B1 at the mRNA level, whereas a 28% increase was observed at the protein level in the M2 differentiated macrophages compared to the undifferentiated control cells (M0) (Figure S3B,D). To further substantiate our data, we carried out immunofluorescence staining with CD163 and AKR1B1 antibodies in FFPE tissues obtained from colon cancer patients. The data showed a clear colocalization of CD163 and AKR1B1 in macrophages both localized in the subepithelial portion of lamina propria in nonneoplastic colon mucosae and tumor stroma of colorectal adenocarcinoma (Figure S4). In colon adenocarcinoma, most of the CD163 positive cells were also positively stained with AKR1B1. These results strongly support our hypothesis that AKR1B1 can be expressed by stromal and epithelial cells, with stronger expression from specific stromal cells such as M2 macrophages.

### 3.3 Analysis of Single Cell RNA‐Seq Data From Colon Tumors

**FIGURE 4:**
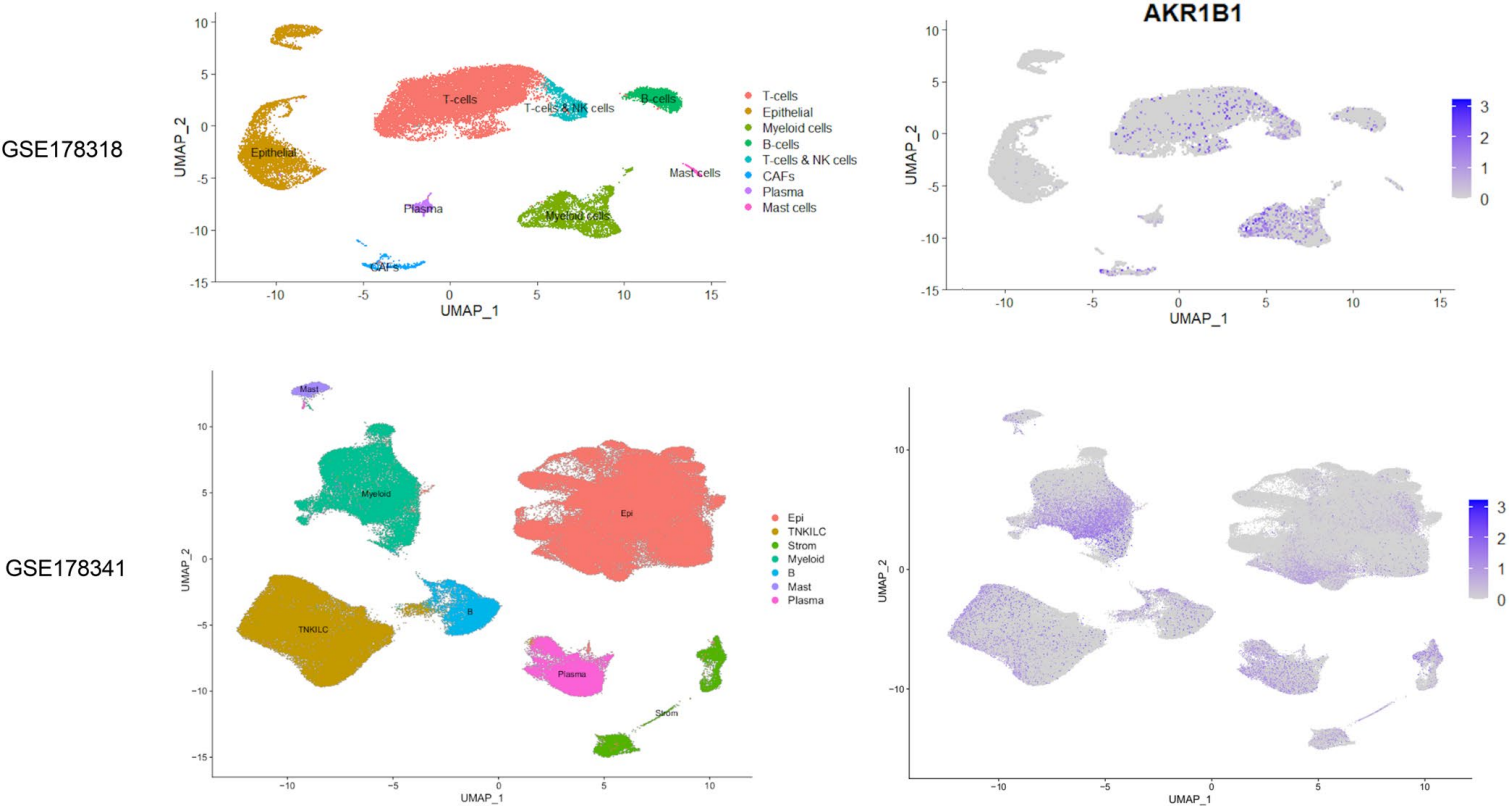
AKR1B1 expression in scRNA‐seq data from CRC tumors in GSE178318 and GSE178341. UMAP plots show the projection of single‐cell transcriptomes onto two dimensions (UMAP‐1 and UMAP‐2), where cells are positioned based on transcriptional similarity. Tumors of three patients that were treatment naive were included in the analysis of GSE178318. Feature plots are shown for cell types (left), and AKR1B1 (right). Mast, Mast cell; Strom, stroma; TNKILC, T and NK and innate lymphoid cells.

In order to better establish the specific cell types that express AKR1B1, we next analyzed scRNA‐seq data obtained from CRC tumors. We observed that AKR1B1 was expressed by myeloid cells, CAFs, and specific subsets of T and NK cells in the TME (Figure 4, Figure S5A). Modest expression of AKR1B1 was also noted in mast cells, plasma, and B cells in GSE178341 (Figure 4, Figure S5B). Strikingly, no AKR1B1 expression in epithelial cells was noted in the dataset GSE178318, whereas in GSE178341 the epithelial expression of AKR1B1 was lower compared to all other cell types tested (Figure S5A,B). These data further support that most of the AKR1B1 expression can be seen in stromal and immune cells in the TME.

CAFs are known to be strongly implicated in tumor progression [19]. As we observed AKR1B1 expression in CAFs (Figure 1B, Figure S5A), we further aimed to evaluate this finding in a larger cohort of colon tumors (GSE39582). Pearson correlation analysis indicated that the expression of six different CAF markers was all positively correlated with the expression of AKR1B1 (*p* < 0.001) (Figure S6); five of these markers had Pearson *r* values above 0.40. Overall, our data suggest that tumors with high CAF content had higher expression of AKR1B1, which is in good agreement with the FACS sorted transcriptomic data of CRC tumors (Figure 1B). When AKR1B1 expression was evaluated in previously published subgroups of CRC based on CAF marker expression [20], AKR1B1 expression was lowest in group 1 (lowest CAF) and gradually increased in groups 2, 3, and 4 (highest CAF) (Figure S6G).

We next analyzed an independent scRNA‐seq dataset (GSE146771) in which data from FACS enriched cell types in colorectal tumors, including subsets of macrophages in the myeloid lineage are available [15]. For all analyses, we used expression data obtained with the Smart‐seq2 technique from 10 CRC tumors. We observed that among all cell types tested, AKR1B1 expression was the highest in myeloid cells (Figure S5C). Expression of AKR1B1 was also noted in B cells, fibroblasts, innate lymphoid cells and CD8 T cells (Figure S5C). In depth analysis of the different cell types identified within the myeloid lineage revealed that AKR1B1 expression was the highest in tumor associated macrophages (TAMs) that express C1QC (complement protein C1Q) gene (Figure S5D). AKR1B1 expression was clearly absent from SPP1 (Secreted Phosphoprotein 1) expressing TAMs. We also noted high AKR1B1 expression in dendritic cells with CD1C and BATF3 expression (conventional dendritic cell 2 [cDC2] and cDC1 cells), respectively (Figure S5D).

### 3.4 Evaluation of AKR1B1 Expression in Colon Adenocarcinoma by IHC and Its Role in Prognosis

**FIGURE 5:**
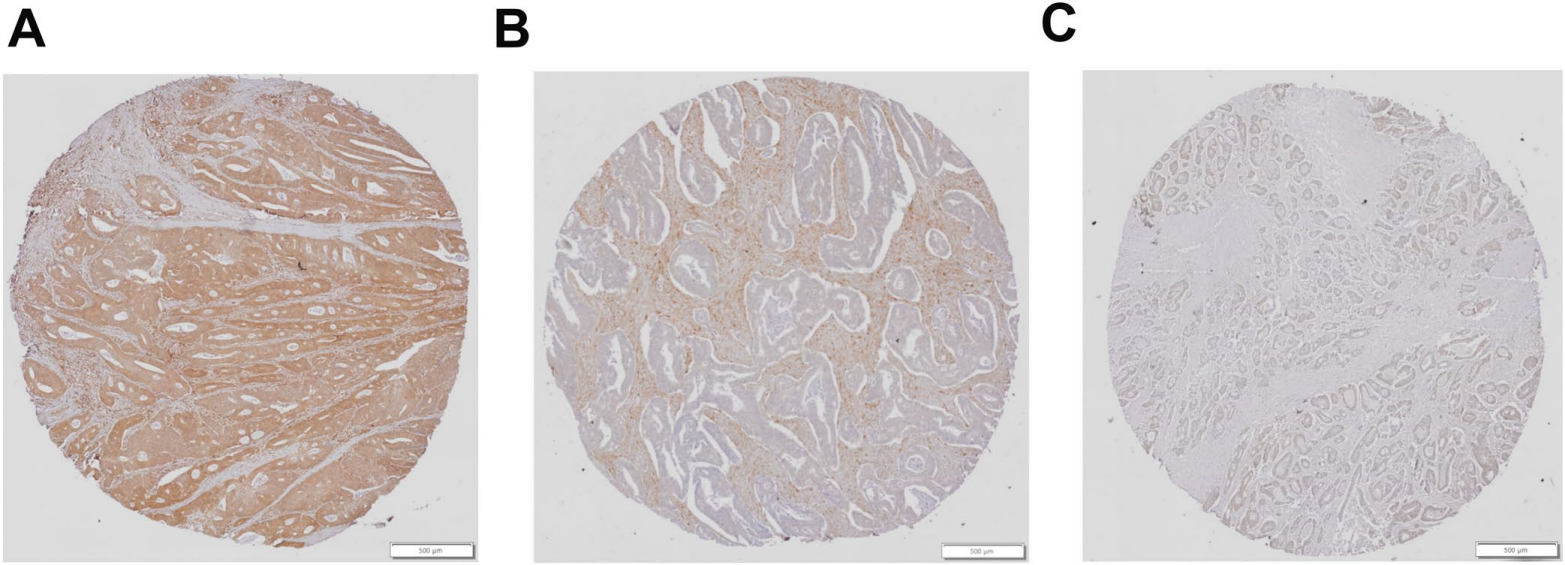
Representative patterns of AKR1B1 expression in colon adenocarcinoma by immunohistochemistry. Neoplastic epithelial cells showed were positively stained for AKR1B1 (A). The predominantly stromal component of the tumor was positively stained for AKR1B1 whereas neoplastic epithelial cells were negatively stained (B). Both epithelial and stromal components of the tumor were negatively stained for AKR1B1 (C).

We next determined the expression of AKR1B1 in the CRC TME by IHC in FFPE tissues obtained from a Turkish cohort. We noted sections with AKR1B1 positivity in neoplastic cells only, stroma only, and sections that had no AKR1B1 expression in either epithelial or stromal compartments, suggesting heterogeneity (Figure 5). Most sections had positive staining in both epithelial and stromal cells at varying intensities. We noted cases that lacked overall AKR1B1 staining, lacked staining in stroma, as well as weak staining in mucinous and signet ring carcinomas (data not shown). Stromal cells, primarily subepithelial macrophages, were seen to stain positively for AKR1B1 in non‐neoplastic colon mucosa (Figure S7, Figure S4), whereas the stroma of colon adenocarcinoma had widespread positivity (Figure S7). Based on the staining patterns, AKR1B1 scores generated from the tumor (tumor AKR1B1 score) and stroma (stromal AKR1B1 score) were evaluated in parallel with the level of stromal infiltration in the tumor core. A higher stromal ratio in the tumor was associated with worse survival (*p*: 0.013) (Figure S8A). Of note, the tumor AKR1B1 score was not associated with either RFS or OS in the Turkish cohort (Figure S8B,C). Surprisingly, the stromal AKR1B1 score was significantly associated with favorable OS in the Turkish cohort (*p*: 0.012) (Figure S8D), along with a borderline nonsignificant association (*p*: 0.065) with RFS (Figure S8E).

Tumors with a higher stromal AKR1B1 score also showed a significantly higher tumor AKR1B1 score (*p* < 0.0001) (Figure S9A). Although these data suggest common mechanisms for the expression of AKR1B1 in both epithelial and stromal compartments, we also observed that the tumor AKR1B1 score was significantly lower in stage 3 tumors (*p* < 0.01, Figure S9B), whereas the stromal AKR1B1 score did not show any significant difference between the different stages (data not shown). Thus, both epithelial and stromal compartments express the protein, but the regulatory pathways leading to the expression may be distinct. We also did not observe any significant relationship between the tumor AKR1B1 score and vascular or lymphatic invasion (Figure S9C,D). The tumor AKR1B1 score was significantly higher in proximal tumors compared to distal tumors (*p*: 0.046, Turkish cohort, Figure S9E) and in mucinous tumors as compared to nonmucinous tumors with borderline significance (*p*: 0.056, Serbian cohort, Figure S9F), which may be due to the presence of mucinous tumors more often in the proximal colon [21]. Analysis of the stromal AKR1B1 score within distal and proximal tumors showed that the stromal AKR1B1 score was associated with good OS in tumors located in the distal colon but not in the proximal colon (Figure S10). These data suggest heterogeneity in the expression of AKR1B1 in the tumor stroma and neoplastic cells, and that the expression from cancer cells was higher in the proximal and mucinous subgroups. In addition, a higher protein level expression of AKR1B1 in the tumor stroma was associated with better prognosis, whereas expression from neoplastic epithelial cells had no significant relationship with the clinical outcome.

We have previously shown that microarray‐based expression of AKR1B1 was associated with poor RFS [4]. When OS was used as the measure of clinical outcome, reanalysis of the GSE39582 dataset indicated that higher AKR1B1 expression was associated with better OS (Figure S11A). In GSE17536, high AKR1B1 expression was associated with poor Disease‐Free Survival (DFS) confirming our findings with RFS in GSE39582 (Figure S11B); however, no significant relationship was identified with Disease Specific Survival (DSS) (Figure S11C). These data suggest high variations in the relationship between AKR1B1 expression and clinical outcomes among different cohorts and with different measures of clinical outcome when evaluated on the basis of bulk expression data from the tumor.

Since we showed that expression of AKR1B1 from tumor stroma was associated with favorable prognosis via IHC, while bulk transcriptomic data suggested the opposite pattern in certain cohorts, we hypothesized that tumor purity may affect clinical outcomes based on AKR1B1 expression. Therefore, we next evaluated prognostic relationships of AKR1B1 based on tumor purity scores generated by the ESTIMATE method. Interestingly, AKR1B1 was associated with poor RFS when the tumor purity was higher (i.e., more epithelial rather than stromal content) (Figure S12). On the other hand, AKR1B1 expression was strongly associated with good OS when the tumor purity was lower (i.e., more stromal content) (Figure S12). Strikingly, significance with OS was lost in tumors with high tumor purity, emphasizing the effect of TME content and the cell types expressing AKR1B1 in AKR1B1's prognostic relationships. Since we observed that AKR1B1 can be expressed by many different cell types in the TME, we next investigated prognostic relationships of AKR1B1 in CRC subgroups defined by the abundance of immune and stromal cells. Noticeably, AKR1B1 expression was associated with favorable prognosis in the CRC subgroups with high stroma and immune infiltration, whereas no significance was noted in subgroups with lower abundance of these cell types (Figure S13). These data suggest that patients with high AKR1B1 expression in a TME with higher stroma and immune content had more favorable prognosis. In order to substantiate these findings at the protein level, we analyzed stromal content based on the H&E stained slides in the Turkish cohort. Tumor samples were classified as “high” and “low” stroma based on the median stromal percentage of the cohort (20%). We observed that stromal B1 score was associated with longer OS in tumors with higher stromal percentage, whereas this significance was lost in tumors with low stromal percentage, supporting our in silico findings (Figure S14).

Overall, our data show for the first time that CRC tumors have expression of AKR1B1 in both epithelial and stromal compartments; moreover, the relationship of AKR1B1 expression and clinical outcome depends highly on the TME content.

## 4 DISSCUSSION

Several different transcriptome‐based systems [16, 22, 23] that have been used for the classification of CRC tumors into different molecular subtypes agree that CRC tumors with high expression of stem cell markers or mesenchymal gene expression show worse prognosis. Further studies have revealed that sole evaluation of epithelial gene expression is insufficient to accurately define CRC molecular subtypes [24]. In fact, a role of stromal activation of mesenchymal signaling was strongly implicated in the assignment of worse prognosis in CRC [24, 25].

Colorectal tumors are generally infiltrated by macrophages, followed by T and B cells; however, most tumors appear to show low infiltration of lymphocytes. Those CRC tumors that have lymphocyte infiltration and high densities of endothelial cells and fibroblasts tend to show worse prognosis [26].

We have previously reported that CRC cell lines expressing AKR1B1 showed increased in vitro migration and proliferation [4]. We and others have also reported the activation of inflammatory signaling in AKR1B1 overexpressing CRC cell lines and tumors [4] as well as in mouse models of colitis with a high expression of AKR1B1 [7]. We have reported the highest level of AKR1B1 expression in CMS4 tumors that are known to be highly mesenchymal, aggressive, rich in stroma, and have active TGFβ signaling, angiogenesis, and inflammation [16]. In addition, we noted high AKR1B1 expression in CMS1 tumors, which show MSI as well as increased immune signaling, along with strong activation of immunosuppressive pathways [16]. These two CMS types are known to be rich in either desmoplastic stroma or immune cells in the TME; thus, this led us to hypothesize that the stromal compartment of CRC tumors may contribute toward oncogenic signaling in AKR1B1 expressing tumors.

Extensive bioinformatics analyses using bulk RNA and scRNA‐seq data strongly indicated that AKR1B1 was expressed in both stromal as well as epithelial cells in CRC, which was validated with IHC. In particular, we observed a strong correlation between AKR1B1 expression and the involvement of M2 macrophages in the TME from bulk transcriptomic data. We also confirmed the expression of AKR1B1 in M2 macrophages in human adenocarcinoma samples and in M2 macrophages differentiated in vitro from THP1 cells. M2 macrophages are generally associated with angiogenesis and tissue repair. Tumor‐associated macrophages (TAM) found in the TME are known to have characteristics similar to M2 macrophages. The presence of TAMs is generally associated with an immune‐suppressive environment, with the recruitment of regulatory T cells and interruption of immune cell interactions and worse prognosis due to the development of EMT and drug resistance [17]. Based on strong correlations between AKR1B1 expression and the fraction of M2 macrophages in the TME, we therefore expected to observe poor prognosis in patients with high stromal expression of AKR1B1. We observed that when tumor purity was high (i.e., tumors had low stromal infiltration), and most of the AKR1B1 expression was contributed by the neoplastic epithelial cells, high expression of AKR1B1 indeed was associated with worse prognosis. The lack of stroma in the in vitro CRC models and previous data from our group indicating an increase in migration and proliferation in CRC cell lines overexpressing AKR1B1 [4] suggests a clear relationship between AKR1B1 expression and aggressive traits and poor prognosis in a low‐stroma TME.

In the current study, we have observed that the expression of AKR1B1 can be contributed by both stromal and epithelial cells in tumors with a high stromal component, and high protein level expression of AKR1B1 from the stroma was associated with favorable prognosis. When tumors were stratified based on their tumor purity, immune and stromal contents, microarray‐based AKR1B1 expression was associated with good OS in tumors with high stroma and immune fractions and low tumor purity. Ex vivo data confirmed that the stromal expression of AKR1B1 was associated with good prognosis specifically in tumors with a higher stromal percentage but not in tumors with a low stromal percentage. These findings overall support that the AKR1B1 and prognosis relationship is highly dependent on the TME and the cell types that express AKR1B1.

To our knowledge, this is the first evidence of the contribution of TME to the relationship between AKR1B1 expression and favorable prognosis. We can suggest several different reasons for our findings. First, there are numerous cell types in the TME; we were able to confirm the expression of AKR1B1 in CD163^+^ myeloid cells. There is remarkable disparity in the literature regarding the role of macrophages in the TME of tumors; nonetheless, several studies suggest improved survival with the infiltration of macrophages in CRC [27]. However, in most of these studies, the macrophages were identified by one or two markers, which may be insufficient to characterize the vast spectrum of macrophages between the M1 and M2 extremes and their role in the TME. CRC with CD163^+^ macrophage infiltration was shown to have high infiltration of immature dendritic cells (DC) and better prognosis, especially in limited disease (UICC I and II) rather than in advanced disease (UICC III and IV) [28]. The stage of the disease may therefore be important in evaluating the role of specific cells infiltrating the TME. Our bioinformatics analyses also suggest that conventional DCs (cDCs) can express AKR1B1. Although this was not experimentally verified in the current study, we speculate that AKR1B1 expression in cDCs may alter the way the TME supports tumor progression. The location of the macrophage can also be important; thus, the presence of macrophages at the invasive front was shown to decrease synchronous and metachronous hepatic metastases [29]. Interestingly, a high macrophage to epithelial cell ratio was shown to improve prognosis in CRC; the same study showed that direct in vitro cell–cell contact between HCT‐116 CRC cells and macrophages led to epithelial cell death, whereas the lack of direct contact (translated to tumors with low macrophage infiltration) led to enhanced cancer cell migration and nuclear beta catenin levels [30].

scRNA‐seq data of CRC tumors revealed the presence of AKR1B1 transcripts in other cell types in the TME as well, suggesting that the combinatorial effect of the expression of AKR1B1 from different cell types as well as the chemokines/cytokines they release may contribute toward prognosis [27]. Thus, the role of AKR1B1 protein and the mechanisms that are associated with a higher AKR1B1 expression in each of these cell types may have an impact on its relationship with clinical outcomes. For example, Shimizu et al. reported that upon tyrosine phosphorylation, AKR1B1 in T cells undergoes significant alterations due to cell‐to‐cell interaction with immunosuppressive macrophages, resulting in the downregulation of T cell functions by suppressor macrophages [11]. The extent of AKR1B1 expression in the numerous different cell types available in the TME and its role in modulating the functions of these cells is currently unknown. An elevated flow of glucose through glycolysis and the pentose phosphate pathway, compared to mitochondrial oxidation, is known to be essential for the inflammatory functions of inflammatory myeloid cells [31]. The reduction of glucose via AKR1B1 leads to the formation of sorbitol and then to fructose [3]. Fructose, even when generated in small amounts, was shown to change the polarization of macrophages from M1 to M2 [32]. It is possible that the source of fructose in the macrophages could have been through the enzymatic activity of AKR1B1. It is also feasible to suggest that AKR1B1 may modulate glucose metabolism and thereby play a role in the inflammatory functions in the TME. This is supported by our previous observation that AKR1B1 expressing tumors had higher expression of pro‐inflammatory cytokines [4]. Future studies including modulation of AKR1B1 expression in various myeloid cell types and coculture of these cells with CRC cells and investigation of the metabolic and transcriptomic changes might provide a deeper understanding of the mechanisms behind the current findings.

In our study, in addition to the expression of AKR1B1 in macrophages, we identified CAFs as another source of AKR1B1 expression. CAFs do not constitute a homogeneous cell population; instead, recent studies have defined specific CAF subpopulations which have different origins, marker proteins, and are known to either promote or suppress cancer progression [33]. It is currently unknown which CAF subtype/subtypes express AKR1B1 among previously defined myofibroblastic CAF (myCAFs), inflammatory CAF (iCAFs), and antigen‐presenting CAF (apCAFs) subtypes, thus both the expression and potential role of AKR1B1 in these cells need to be elucidated.

The current study has some limitations that need to be specified. The immunohistochemistry relied on tissue microarrays, which provide data on a very small region of the tumor. Considering tumor heterogeneity, future studies can include samples that are collected from different regions of the same tumor. Although AKR1B1 expression was strongly correlated with the expression of multiple macrophage markers in silico, validation of coexpression was performed using only one macrophage marker. However, macrophages have numerous markers and can belong to several different subtypes. A better classification of which macrophage expresses AKR1B1 most robustly needs to be identified. We did not experimentally verify the expression of AKR1B1 in CAFs in the current study. Thus, future experiments can be designed to determine whether FAP positive fibroblasts also express AKR1B1.

In summary, in the current study we evaluated the expression and prognostic significance of AKR1B1 in CRC, focusing on its presence within the TME. Based on analyses of multiple transcriptomic datasets, we showed that AKR1B1 is highly expressed in the tumor stroma, specifically in CAFs and myeloid cells, compared to relatively lower expression in epithelial cells. IF‐based double staining experiments using colon adenocarcinoma tissues confirmed that AKR1B1 was indeed expressed by CD163 positive M2 macrophages. In vitro, M2 macrophages differentiated from THP1 cells showed AKR1B1 expression. Microarray‐based prognostic analyses revealed that high AKR1B1 expression was associated with favorable OS in tumors with low tumor purity and high stromal or immune infiltration. In line with these findings, stromal AKR1B1 score based on IHC stainings was significantly associated with favorable OS in the Turkish cohort. Thus, these data highlight that the prognostic associations of AKR1B1 i highly dependent on the context of the TME, and that the stromal expression of AKR1B1 may have a role in CRC progression. Overall, the study underscores the importance of characterizing the specific cell populations within the TME that express potential biomarkers, such as AKR1B1, to improve the interpretation of gene expression‐based biomarkers in CRC.

## Author Contributions

S.D.C. and A.N. obtained funding for the study with contributions from S.B. S.D.C. administered the project. S.D.C., A.N., and S.B. contributed to the design and conceptualization of the study. S.B., A.N., A.A., and S.D.C. contributed to the investigation and supervision. S.D.C., E.G.S., A.I., B.S.‐J., A.D.R., G.G., Ö.D., and S.D. contributed to data curation. S.D.C., E.G.S., A.I., B.S.‐J., G.G., A.D.R., and S.D. contributed to methodology and formal analyses. S.D.C. and S.B. contributed to writing – original draft. S.D.C., S.B., E.G.S., A.A., B.S.‐J., Ö.D., G.G., and A.N. contributed to writing – review and editing.

## Disclosure

The authors have nothing to report.

## Ethics Statement

Ethical approval for the Turkish cohort was obtained from the Noninvasive Clinical Researches Ethics Board of Hacettepe University (project number: GO 20/1184, approval number: 2020/20‐63, December 15, 2020). Ethical approval for the surgical samples and FFPE samples from the Serbian cohort was obtained from the Ethical Committee of the Faculty of Medicine, University of Belgrade (No: 1550/V‐2, May 31, 2019) and from the Ethical Committee of the University Clinical Center of Serbia (No: 175/1, April 27, 2021), respectively.

## Conflicts of Interest

The authors declare no conflicts of interest.

## Supporting information


**Figure S1.** AKR1B1 expression in tumor and adjacent normal tissues and across CMS subtypes.
**Figure S2**. Expression of AKR1B1 in the macrophages.
**Figure S3**. AKR1B1 expression upon M2 macrophage differentiation. TGM2 and CD163 were used as markers of M2 macrophages.
**Figure S4**. Immunoflourescence staining of AKR1B1 and CD163 in colon adenocarcinoma and normal mucosa from the Turkish colon cancer patients.
**Figure S5**. AKR1B1 expression in scRNA‐seq data from CRC tumors.
**Figure S6**. Expression of AKR1B1 and CAF markers in CRC.

**Figure S7.** Representative staining patterns with AKR1B1 from colon mucosa and adenocarcinoma of the Turkish patients.

**Figure S8.** Evaluation of prognostic relationships in the Turkish CRC cohort.
**Figure S9**. Tumor AKR1B1 score in tumors with different clinical characteristics.
**Figure S10**. Kaplan Meier graphs of stromal AKR1B1 score.
**Figure S11**. Log rank multiple cutoff graphs for AKR1B1 expression.
**Figure S12**. Log rank multiple cutoff graphs for AKR1B1 expression stratified by tumor purity in GSE39582.
**Figure S13**. Log rank multiple cutoff graphs for AKR1B1 expression stratified by ESTIMATE scores in GSE39582.
**Figure S14**. Kaplan Meier graphs of stromal AKR1B1 score.

**Appendix S1.** Supplementary materials and methods.

**Table S1.** Primer pairs used in the study.

## Data Availability

The transcriptomic datasets analyzed in this study are accessible through public databases, as detailed in the methods section. Detailed materials and protocols are available in the Supporting Information section of this paper. Data generated from the ex vivo cohorts are available from the corresponding author upon reasonable request.

## References

[1] J. Li , D. Chen , and M. Shen , “Tumor Microenvironment Shapes Colorectal Cancer Progression, Metastasis, and Treatment Responses,” Frontiers in Medicine 9 (2022): 869010. 35402443 10.3389/fmed.2022.869010 PMC8984105

[2] T. M. Penning , “The Aldo‐Keto Reductases (AKRs): Overview,” Chemico‐Biological Interactions 234 (2015): 236–246. 25304492 10.1016/j.cbi.2014.09.024 PMC4388799

[3] S. Banerjee , “Aldo Keto Reductases AKR1B1 and AKR1B10 in Cancer: Molecular Mechanisms and Signaling Networks,” Advances in Experimental Medicine and Biology 1347 (2021): 65–82. 33945128 10.1007/5584_2021_634

[4] B. Taskoparan , E. G. Seza , S. Demirkol , et al., “Opposing Roles of the Aldo‐Keto Reductases AKR1B1 and AKR1B10 in Colorectal Cancer,” Cellular Oncology (Dordrecht) 40, no. 6 (2017): 563–578. 10.1007/s13402-017-0351-7 PMC13001554 28929377

[5] S. Demirkol Canli , E. G. Seza , I. Sheraj , et al., “Evaluation of an Aldo‐Keto Reductase Gene Signature With Prognostic Significance in Colon Cancer via Activation of Epithelial to Mesenchymal Transition and the p70S6K Pathway,” Carcinogenesis 41, no. 9 (2020): 1219–1228. 32628753 10.1093/carcin/bgaa072

[6] L. Liu , L. Zhu , Z. Cheng , Y. Sun , Y. Zhou , and J. Cao , “Aberrant Expression of AKR1B1 Indicates Poor Prognosis and Promotes Gastric Cancer Progression by Regulating the AKT‐mTOR Pathway,” Aging 15, no. 18 (2023): 9661–9675. 37751590 10.18632/aging.205041 PMC10564407

[7] N. P. Syamprasad , B. Rajdev , S. Jain , et al., “Pivotal Role of AKR1B1 in Pathogenesis of Colitis Associated Colorectal Carcinogenesis,” International Immunopharmacology 119 (2023): 110145. 37044030 10.1016/j.intimp.2023.110145

[8] M. Hojnik , S. Frković Grazio , I. Verdenik , and T. L. Rižner , “AKR1B1 and AKR1B10 as Prognostic Biomarkers of Endometrioid Endometrial Carcinomas,” Cancers 13, no. 14 (2021): 3398. 34298614 10.3390/cancers13143398 PMC8305663

[9] X. Wu , X. Li , Q. Fu , et al., “AKR1B1 Promotes Basal‐Like Breast Cancer Progression by a Positive Feedback Loop That Activates the EMT Program,” Journal of Experimental Medicine 214, no. 4 (2017): 1065–1079. 28270406 10.1084/jem.20160903 PMC5379972

[10] Z. Zhao , Z. Hao , Z. Zhang , and X. Zhan , “Bioinformatics Analysis Reveals the Vital Role of AKR1B1 in Immune Infiltration and Clinical Outcomes of Gastric Cancer,” DNA and Cell Biology 42, no. 7 (2023): 372–389. 37285280 10.1089/dna.2022.0644

[11] T. Shimizu , Y. Tatano , and H. Tomioka , “Aldose Reductase Participates in the Downregulation of T Cell Functions due to Suppressor Macrophages,” Scientific Reports 6 (2016): 21093. 26868163 10.1038/srep21093 PMC4751572

[12] A. Akyol , G. Güner , H. S. Özşeker , et al., “An Immunohistochemical Approach to Detect Oncogenic CTNNB1 Mutations in Primary Neoplastic Tissues,” Laboratory Investigation 99, no. 1 (2019): 128–137. 30177831 10.1038/s41374-018-0121-9

[13] S. Demirkol , I. Gomceli , M. Isbilen , et al., “A Combined ULBP2 and SEMA5A Expression Signature as a Prognostic and Predictive Biomarker for Colon Cancer,” Journal of Cancer 8, no. 7 (2017): 1113–1122. 28607584 10.7150/jca.17872 PMC5463424

[14] K. Pelka , M. Hofree , J. H. Chen , et al., “Spatially Organized Multicellular Immune Hubs in Human Colorectal Cancer,” Cell 184, no. 18 (2021): 4734–4752. 34450029 10.1016/j.cell.2021.08.003 PMC8772395

[15] L. Zhang , Z. Li , K. M. Skrzypczynska , et al., “Single‐Cell Analyses Inform Mechanisms of Myeloid‐Targeted Therapies in Colon Cancer,” Cell 181, no. 2 (2020): 442–459. 32302573 10.1016/j.cell.2020.03.048

[16] J. Guinney , R. Dienstmann , X. Wang , et al., “The Consensus Molecular Subtypes of Colorectal Cancer,” Nature Medicine 21, no. 11 (2015): 1350–1356. 10.1038/nm.3967 PMC4636487 26457759

[17] S. Wang , J. Wang , Z. Chen , et al., “Targeting M2‐Like Tumor‐Associated Macrophages Is a Potential Therapeutic Approach to Overcome Antitumor Drug Resistance,” npj Precision Oncology 8, no. 1 (2024): 31. 38341519 10.1038/s41698-024-00522-z PMC10858952

[18] L. He , J. H. Jhong , Q. Chen , et al., “Global Characterization of Macrophage Polarization Mechanisms and Identification of M2‐Type Polarization Inhibitors,” Cell Reports 37, no. 5 (2021): 109955. 34731634 10.1016/j.celrep.2021.109955 PMC8783961

[19] D. Yang , J. Liu , H. Qian , and Q. Zhuang , “Cancer‐Associated Fibroblasts: From Basic Science to Anticancer Therapy,” Experimental & Molecular Medicine 55, no. 7 (2023): 1322–1332. 37394578 10.1038/s12276-023-01013-0 PMC10394065

[20] S. Demirkol Canli , “Evaluation of Prognostic Markers in Cancer‐Associated Fibroblast Based Sub‐Groups of Colorectal Cancer,” Acta Medica 53, no. 2 (2022): 133–143.

[21] C. Luo , S. Cen , G. Ding , and W. Wu , “Mucinous Colorectal Adenocarcinoma: Clinical Pathology and Treatment Options,” Cancer Communications 39, no. 1 (2019): 13. 30922401 10.1186/s40880-019-0361-0 PMC6440160

[22] E. M. F. De Sousa , X. Wang , M. Jansen , et al., “Poor‐Prognosis Colon Cancer Is Defined by a Molecularly Distinct Subtype and Develops From Serrated Precursor Lesions,” Nature Medicine 19, no. 5 (2013): 614–618. 10.1038/nm.3174 23584090

[23] A. Sadanandam , C. A. Lyssiotis , K. Homicsko , et al., “A Colorectal Cancer Classification System That Associates Cellular Phenotype and Responses to Therapy,” Nature Medicine 19, no. 5 (2013): 619–625. 10.1038/nm.3175 PMC3774607 23584089

[24] A. Calon , E. Lonardo , A. Berenguer‐Llergo , et al., “Stromal Gene Expression Defines Poor‐Prognosis Subtypes in Colorectal Cancer,” Nature Genetics 47, no. 4 (2015): 320–329. 25706628 10.1038/ng.3225

[25] J. Kreis , B. Aybey , F. Geist , B. Brors , and E. Staub , “Stromal Signals Dominate Gene Expression Signature Scores That Aim to Describe Cancer Cell‐Intrinsic Stemness or Mesenchymality Characteristics,” Cancer Research Communications 4, no. 2 (2024): 516–529. 38349551 10.1158/2767-9764.CRC-23-0383 PMC10885853

[26] E. Becht , N. A. Giraldo , C. Germain , et al., “Immune Contexture, Immunoscore, and Malignant Cell Molecular Subgroups for Prognostic and Theranostic Classifications of Cancers,” Advances in Immunology 130 (2016): 95–190. 26923001 10.1016/bs.ai.2015.12.002

[27] M. L. Pinto , E. Rios , C. Durães , et al., “The Two Faces of Tumor‐Associated Macrophages and Their Clinical Significance in Colorectal Cancer,” Frontiers in Immunology 10 (2019): 1875. 31481956 10.3389/fimmu.2019.01875 PMC6710360

[28] D. Nagorsen , S. Voigt , E. Berg , H. Stein , E. Thiel , and C. Loddenkemper , “Tumor‐Infiltrating Macrophages and Dendritic Cells in Human Colorectal Cancer: Relation to Local Regulatory T Cells, Systemic T‐Cell Response Against Tumor‐Associated Antigens and Survival,” Journal of Translational Medicine 5 (2007): 62. 18047662 10.1186/1479-5876-5-62 PMC2212626

[29] Q. Zhou , R. Q. Peng , X. J. Wu , et al., “The Density of Macrophages in the Invasive Front Is Inversely Correlated to Liver Metastasis in Colon Cancer,” Journal of Translational Medicine 8 (2010): 13. 20141634 10.1186/1479-5876-8-13 PMC2841127

[30] J. Forssell , A.°. Öberg , M. L. Henriksson , R. Stenling , A. Jung , and R. Palmqvist , “High Macrophage Infiltration Along the Tumor Front Correlates With Improved Survival in Colon Cancer,” Clinical Cancer Research 13, no. 5 (2007): 1472–1479. 17332291 10.1158/1078-0432.CCR-06-2073

[31] T. Nishizawa , J. E. Kanter , F. Kramer , et al., “Testing the Role of Myeloid Cell Glucose Flux in Inflammation and Atherosclerosis,” Cell Reports 7, no. 2 (2014): 356–365. 24726364 10.1016/j.celrep.2014.03.028 PMC4021396

[32] H. Yan , Z. Wang , D. Teng , et al., “Hexokinase 2 Senses Fructose in Tumor‐Associated Macrophages to Promote Colorectal Cancer Growth,” Cell Metabolism 36, no. 11 (2024): 2449–2467. 39471815 10.1016/j.cmet.2024.10.002

[33] Y. Yamamoto , H. Kasashima , Y. Fukui , G. Tsujio , M. Yashiro , and K. Maeda , “The Heterogeneity of Cancer‐Associated Fibroblast Subpopulations: Their Origins, Biomarkers, and Roles in the Tumor Microenvironment,” Cancer Science 114, no. 1 (2023): 16–24. 36197901 10.1111/cas.15609 PMC9807521

