# Supplementary material for: AKR1B1 Expression in the Colorectal Tumor Microenvironment Contributes Towards Its Prognostic Significance
Source: Cancer Med. 2025 May 21;14(10):e70974. doi: 10.1002/cam4.70974 (PMC12093151; doi:10.1002/cam4.70974)

Fig. S1:

Supplementary Figure 1: AKR1B1 expression in tumor and adjacent normal tissues and across CMS subtypes. RT-PCR based quantification of AKR1B1 expression in rectal tumor and adjacent paired normal tissues obtained from Serbian patients (A). AKR1B1 expression in CMS subtypes (GSE39582) (B). Wilcoxon matched-pairs signed rank test (A) and one way ANOVA with Tukey’s multiple comparisons test (B) p values are indicated ** p<0.01,*** p<0.001, ****p<0.0001.


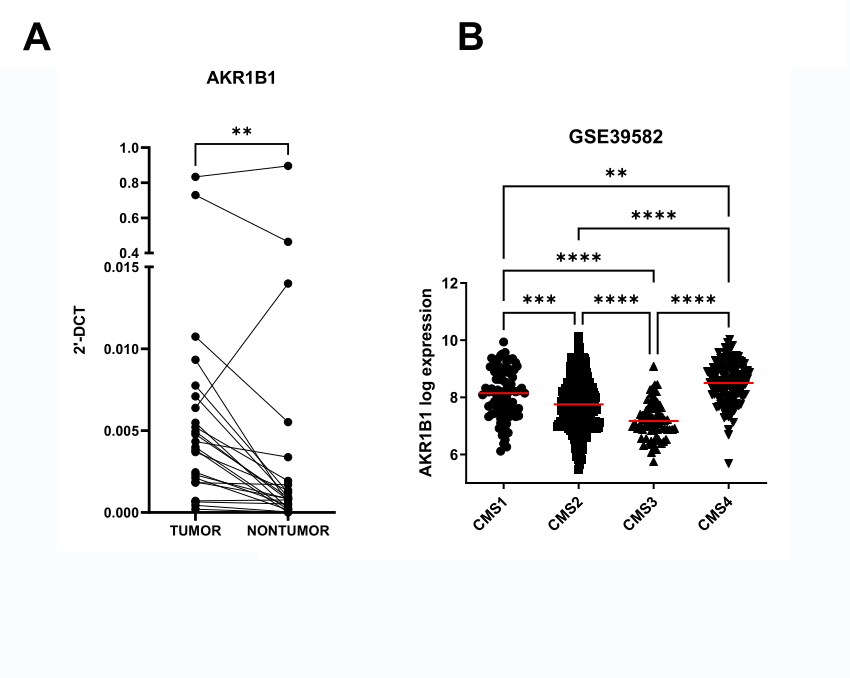


Fig. S2:

Relationship of AKR1B1 expression and macrophages. Correlation of AKR1B1 expression (x-axis) with M1 (A,C) and M2 (B,D) macrophage fractions in GSE39582 (A,B) and GSE17536 (C,D). Pearson r and p values are given. AKR1B1 expression (y-axis) in differentiated M1 and M2 macrophages from naïve macrophages in GSE16385 (E). Labeling of the samples are given as a short abbreviation of replicate number (1 or 2), experimental condition (control or treatment), cell type (macrophage) and the duration of the treatment (12h), e.g. "1 IFNg+TNF macrophage, 12 h". Expression of AKR1B1 (y-axis) in M1 and M2 macrophages differentiated from human PBMCs, GSE117040 (F). Donors are labeled with the letters A, B, C, D.


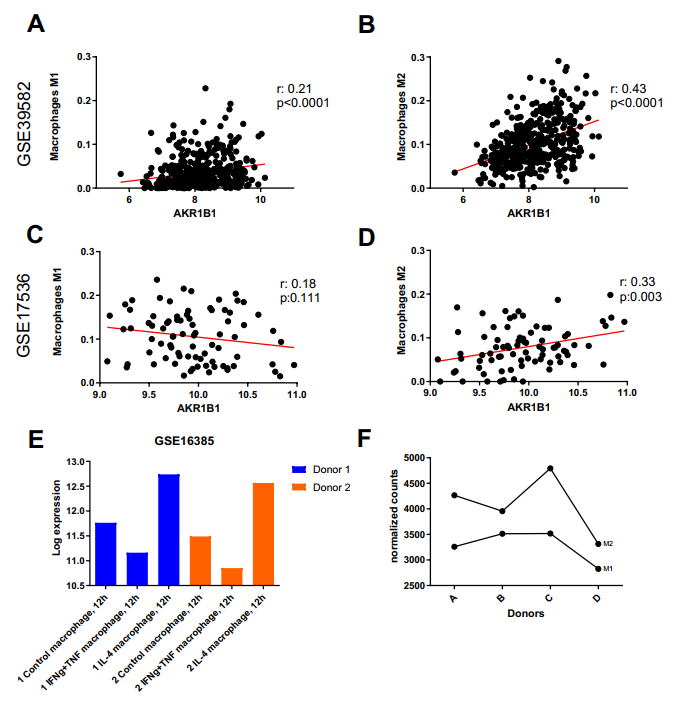


Fig. S3:

AKR1B1 expression upon M2 differentiation. TGM2 and CD163 were used as the markers of M2 macrophages. qPCR based assessment of TGM2 (A) and AKR1B1 (B) expression. M0: Cells cultured with conditioned media after PMA treatment, M2: Cells cultured with IL-4 and IL-13 after PMA treatment. Box and whiskers indicate mean and standard error of mean, respectively. Western blot based quantification of CD163 and AKR1B1 expression (C). Band intensities were calculated as fold changes in reference to α-Tubulin (D). * p<0.05


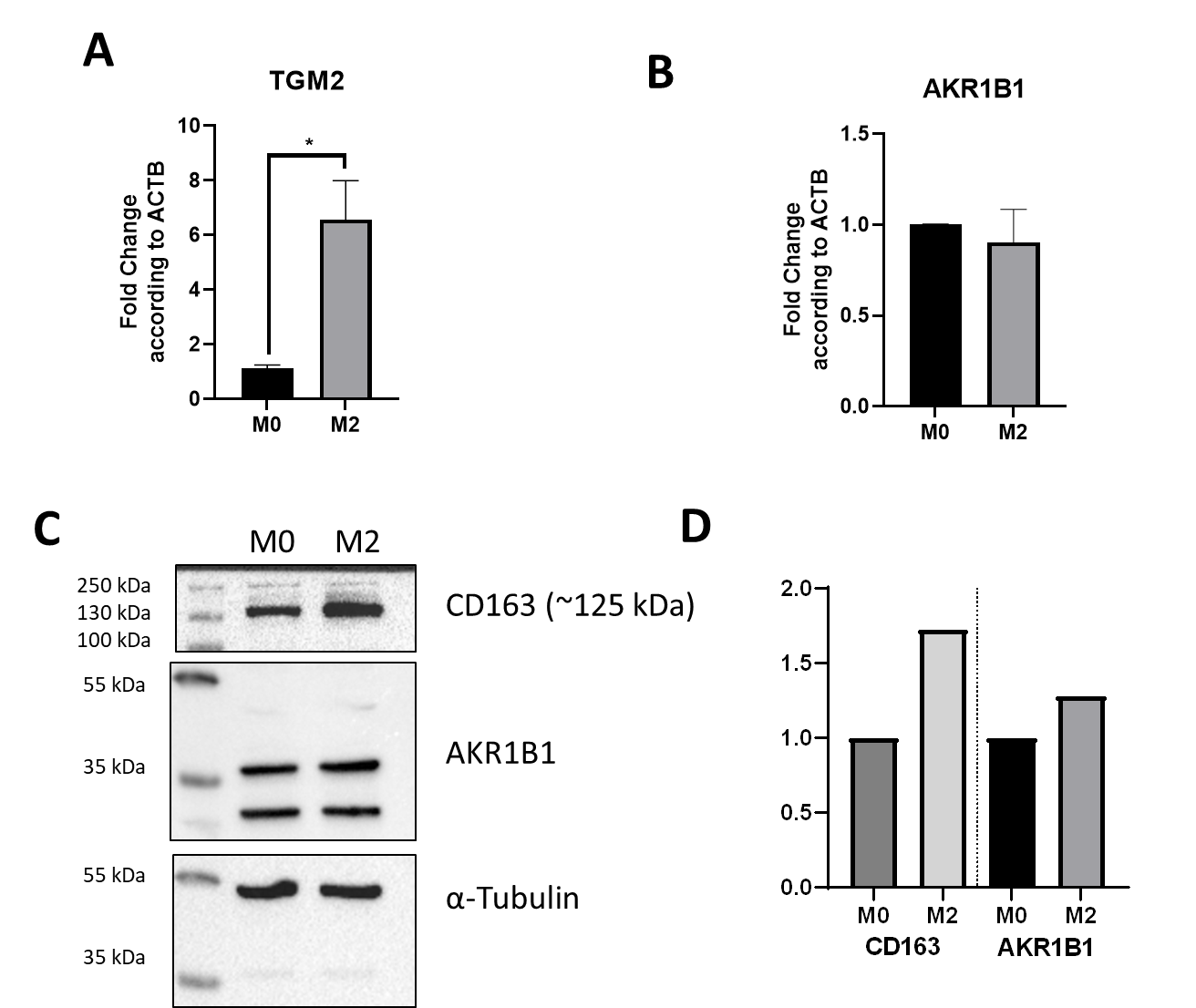


Fig. S4:

Immunoflourescence staining of AKR1B1 and CD163 in colon adenocarcinoma and normal mucosa from the Turkish colon cancer patients. Yellow arrows: Colocalization under the epithelium.


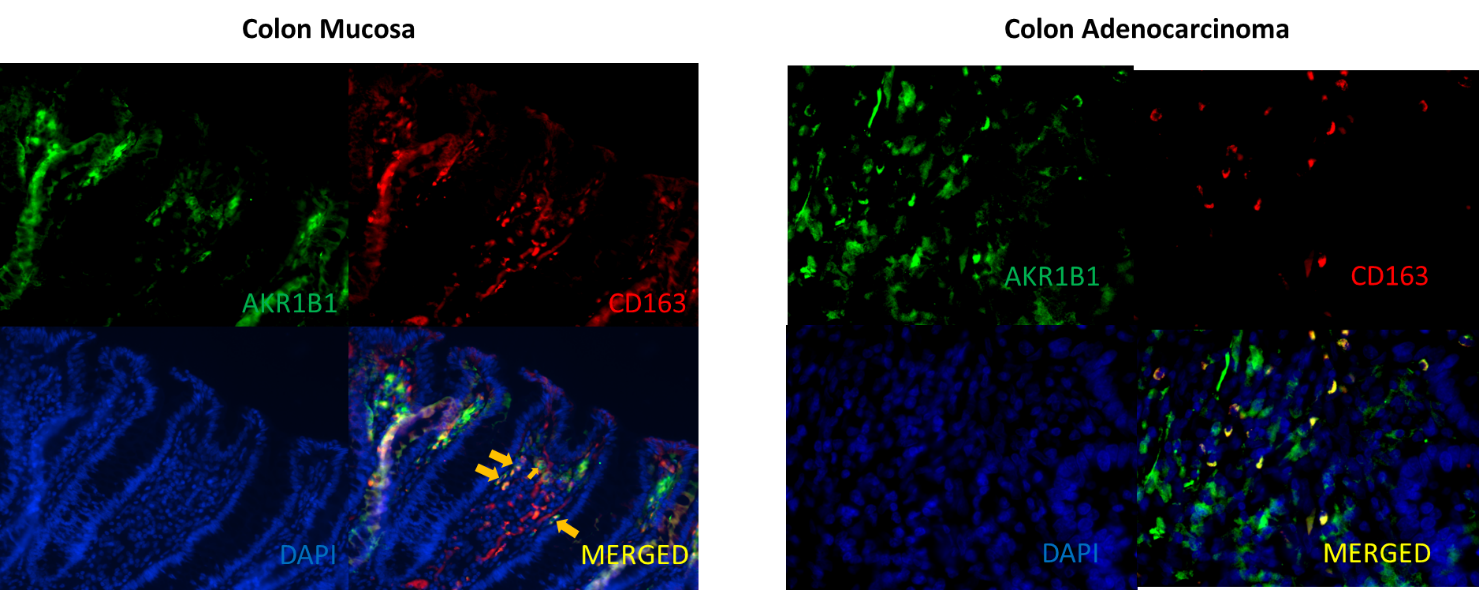


Fig. S5:

AKR1B1 expression in scRNAseq data from CRC tumors. Violin plots for AKR1B1 expression levels shown in Figure 3 for GSE178318 (A), GSE178341 (B). AKR1B1 expression in CRC tumors in GSE146771 (Smart-seq), when all cell types are included (C) and when only myeloid lineage was analyzed (D). ILC: Innate lymphoid cells, TAM:Tumor associated macrophage, Mono: Monocyte, Macro: Macrophage, cDC: conventional dendritic cell, pDC: Plasmacytoid DC, Mast: Mast cell, TNKILC: T and NK and innate lymphoid cells. Myeloid subsets are labeled as hM01: Mast cells, hM02: Plasmacytoid DC, hM03: Conventional dendritic cell 2, hM04: Conventional dendritic cell 1, hM05: Classical CD14^hi^CD16^−^ monocytes, hM06: Non-classical CD14^+^CD16^hi^  monocytes, hM08: Resident tissue macrophages , hM12: CD68^+^ CD80^+^ MAF^+^ Tissue associated macrophages (TAM), hM13: CD68^+^ MARCO^+^ VEGFA^+^ TAMs.


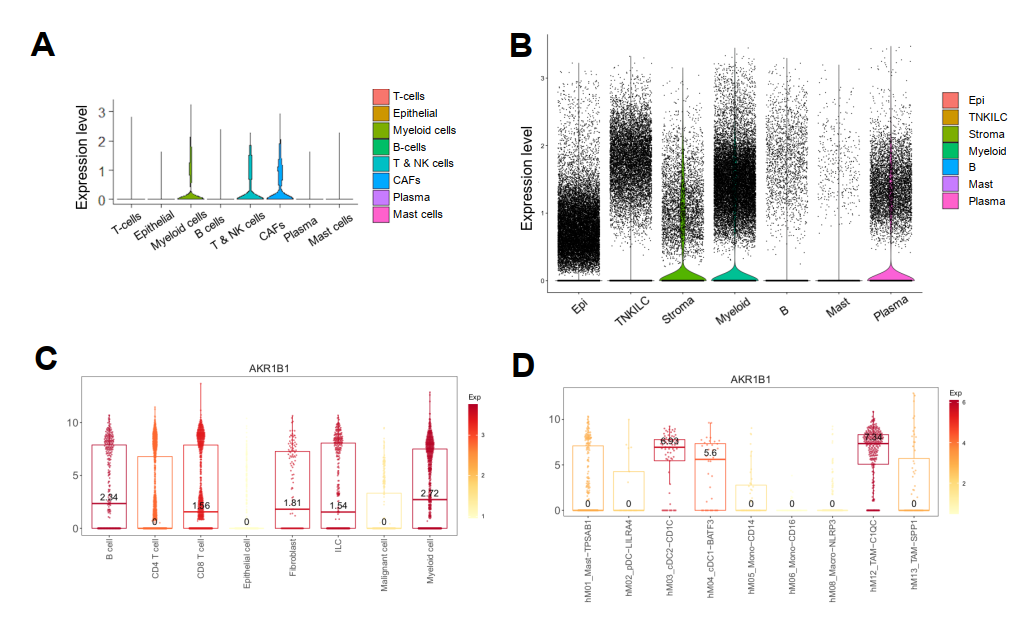


Fig. S6:

Expression of AKR1B1 and CAF markers in CRC. Linear correlation of the expression of CAF markers and AKR1B1 (x-axis) in GSE39582 (A-F). Pearson r and p values are given. Previously published CRC subgroups based on CAF marker expression (x-axis) and AKR1B1 expression (y-axis) in GSE39582 (G). **** p<0.0001


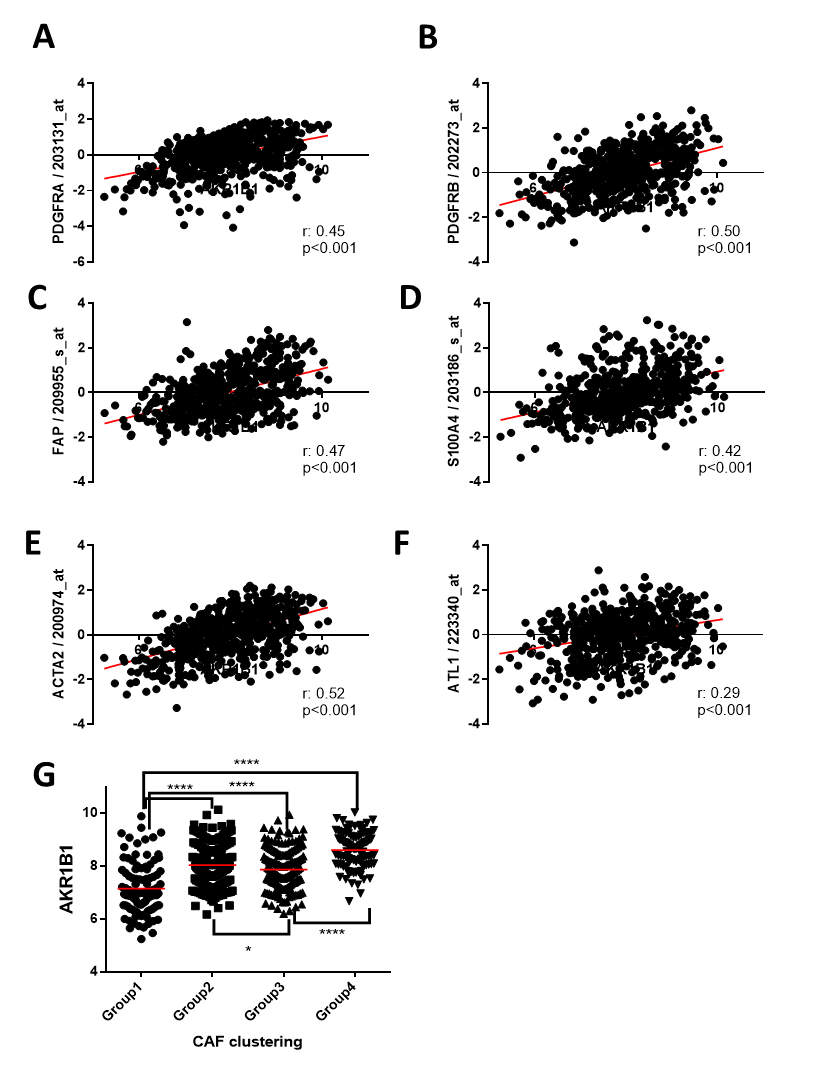

Supplement: Supplementary file 1 — Figure S1. AKR1B1 expression in tumor and adjacent normal tissues and across CMS subtypes. Figure S2. Expression of AKR1B1 in the macrophages. Figure S3. AKR1B1 expression upon M2 macrophage differentiation. TGM2 and CD163 were used as markers of M2 macrophages. Figure S4. Immunoflourescence staining of AKR1B1 and CD163 in colon adenocarcinoma and normal mucosa from the Turkish colon cancer patients. Figure S5. AKR1B1 expression in scRNA‐seq data from CRC tumors. Figure S6. Expression of AKR1B1 and CAF markers in CRC. [file CAM4-14-e70974-s003.docx]
