# Supplementary material for: AKR1B1 Expression in the Colorectal Tumor Microenvironment Contributes Towards Its Prognostic Significance
Source: Cancer Med. 2025 May 21;14(10):e70974. doi: 10.1002/cam4.70974 (PMC12093151; doi:10.1002/cam4.70974)

Supplementary Figure 7:
Representative staining patterns with AKR1B1 from colon mucosa and adenocarcinoma of the Turkish patients. The slides were incubated with the primary antibody (1:800 dilution) for 1 hour at room temprature. The Ultravision Polyvalent (rabbit-mouse) HRP Kit (TP-125-HL, Thermo Scientific) with the avidin-biotin peroxidase method was used.

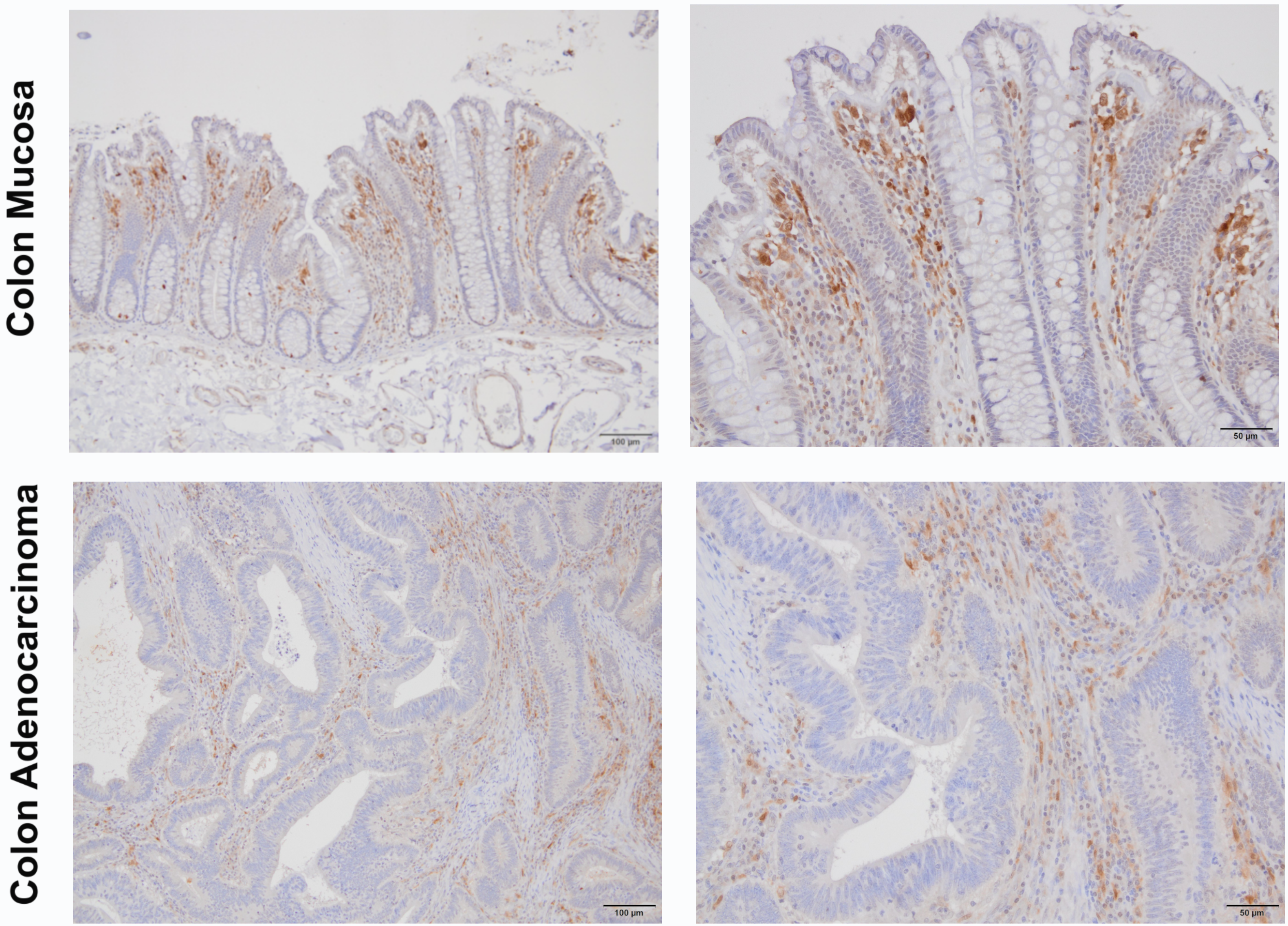

Supplement: Supplementary file 2 — Figure S7. Representative staining patterns with AKR1B1 from colon mucosa and adenocarcinoma of the Turkish patients. [file CAM4-14-e70974-s005.pdf]
