# Supplementary material for: AKR1B1 Expression in the Colorectal Tumor Microenvironment Contributes Towards Its Prognostic Significance
Source: Cancer Med. 2025 May 21;14(10):e70974. doi: 10.1002/cam4.70974 (PMC12093151; doi:10.1002/cam4.70974)

Fig. S8:

Evaluation of prognostic relationships in the Turkish CRC cohort. Stromal ratio in the tumor core (A) is shown. High stroma: Stromal ratio above 40, Low stroma: Stromal ratio equal to or below 40. Log-rank multiple cut-off graphs were shown for tumor B1 score in terms of OS (B) and RFS (C). Horizontal dashed line indicates 0.05 p value. Kaplan Meier graphs are given for stromal B1 score for OS (D) and RFS (E).


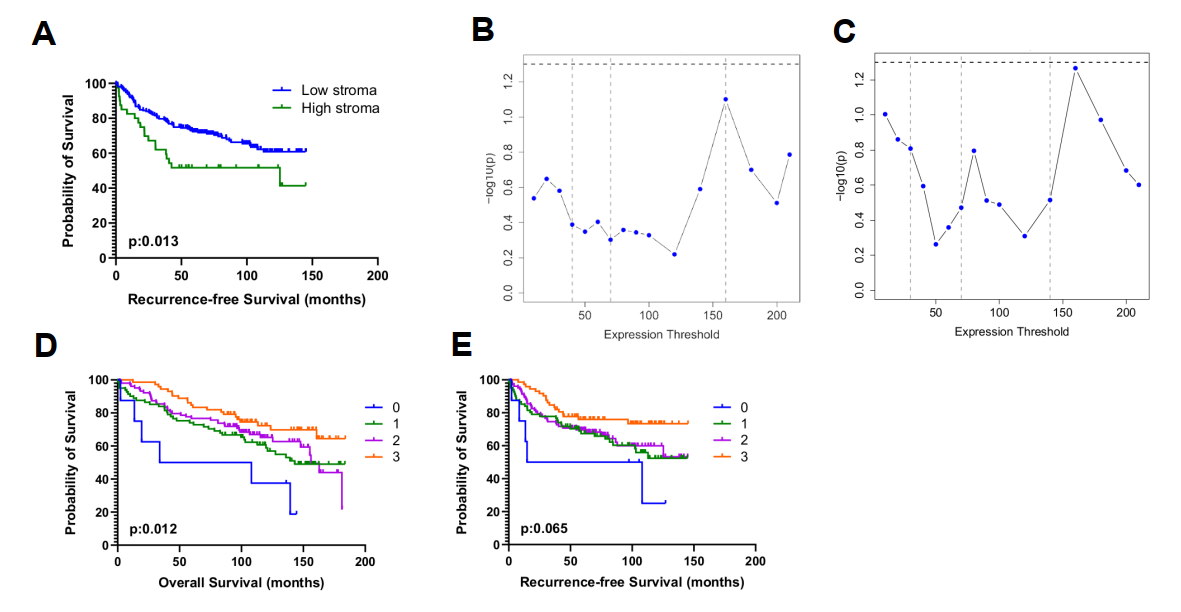


Fig. S9:

Tumor B1 score in tumors with different clinical characteristics. Scores from Turkish cohort (A-E) and Serbian cohort (F). Stromal score: Semi-quantitative score of stromal AKR1B1 expression: no staining (0), mild (1), moderate (2), and strong (3). Low: Stromal score of 0-1, High: Stromal score of 2-3. Box plot shows data between 25th and 75th percentiles. Horizontal line indicates median. Whiskers represent 10-90 percentiles. Data points beyond 10-90 percentiles are shown as dots. Man Whitney test were performed for all comparisons. Muc: mucinuous, nonmuc: nonmucinuous. *p<0.05, **p<0.01, ****p<0.0001, ns: nonsignificant


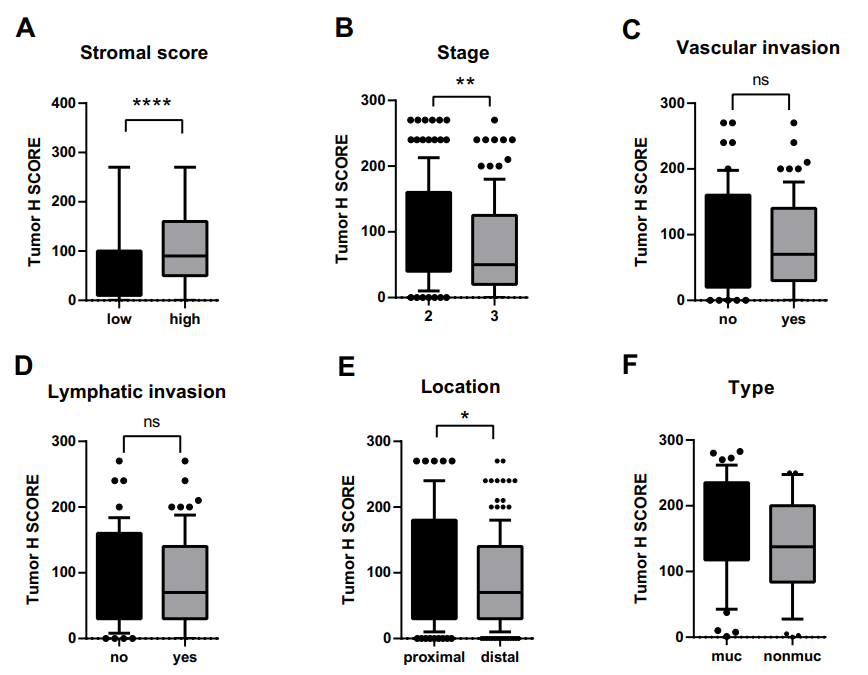


Fig. S10:

Kaplan Meier graphs of stromal B1 score. Patients were stratified based tumor location; proximal (A) and distal (B). Log rank p values are given.


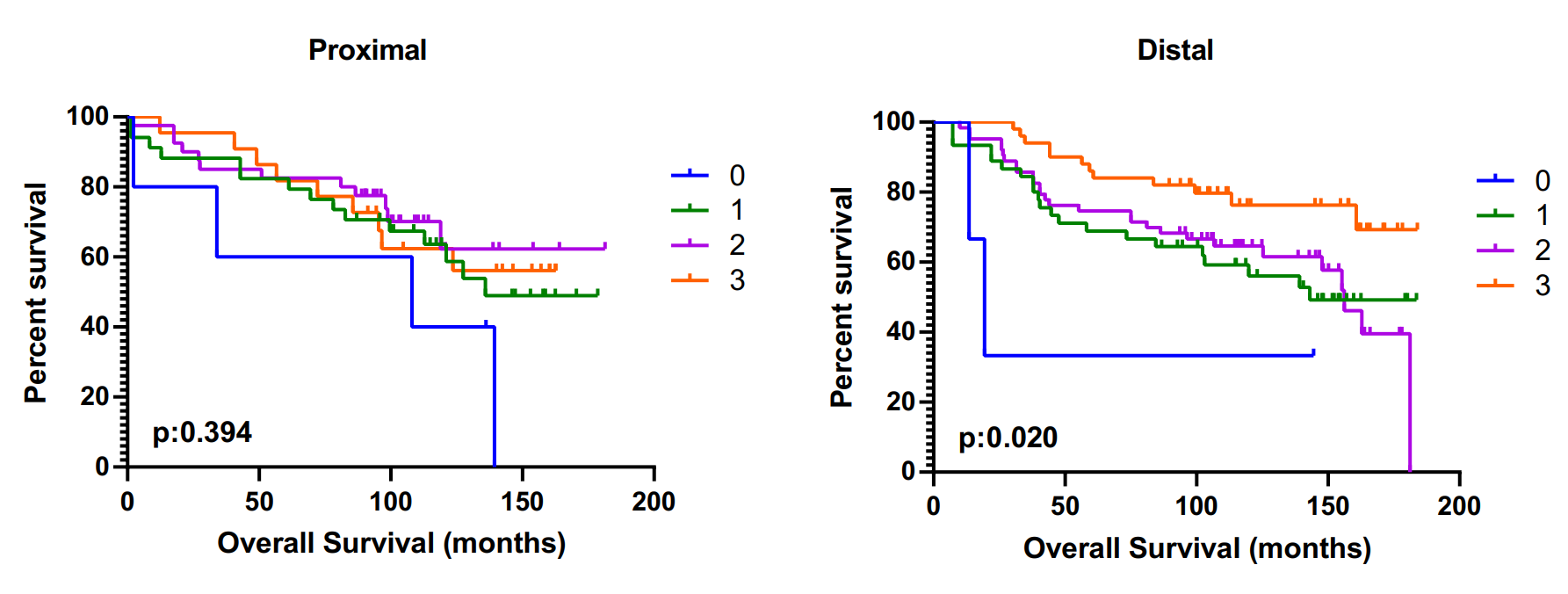


Fig. S11:

Log rank multiple cut-off graphs for AKR1B1 expression. OS was used as clinical outcome in GSE39582 (A). DFS (B) and DSS (C) were used as the patient outcome in GSE17536. Samples with nonzero survival data and available status were included in the analysis.


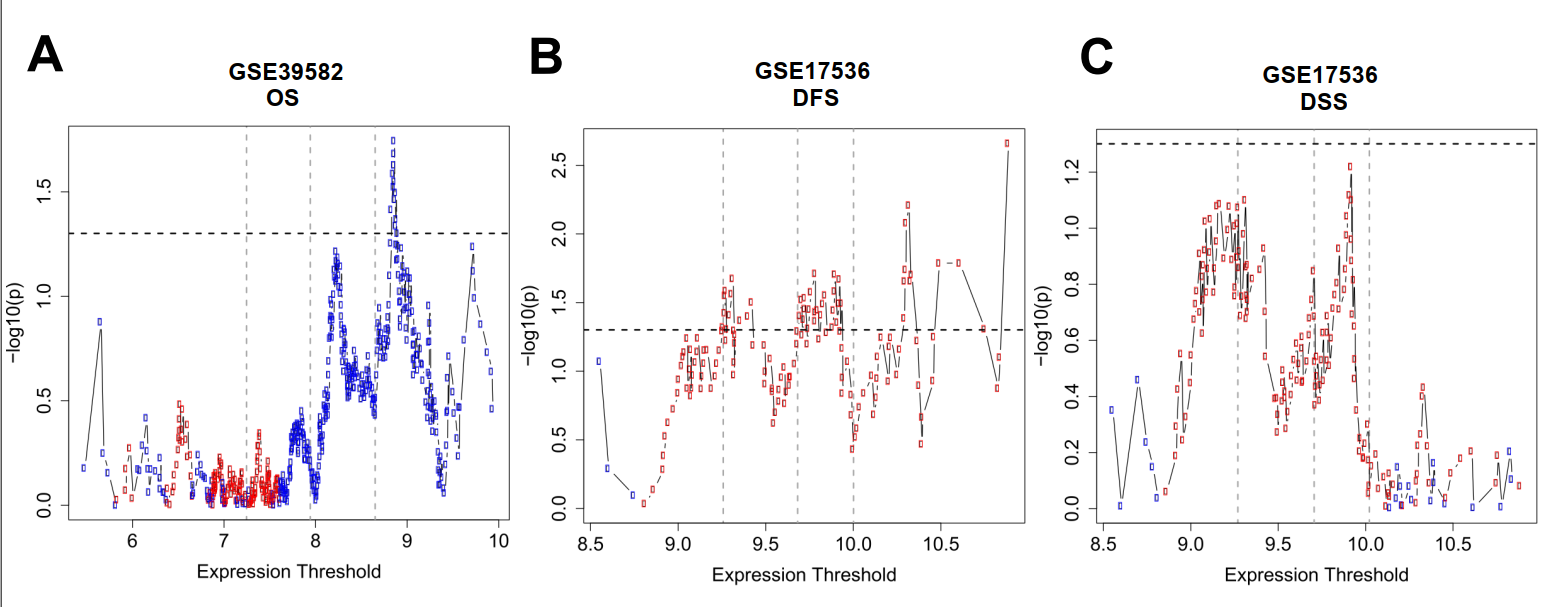


Fig. S12:

Log rank multiple cut-off graphs for AKR1B1 expression stratified by tumor purity in GSE39582. Low: Low tumor purity, Int: Intermediate tumor purity, High: High tumor purity. RFS (upper panel), and OS (lower panel) are used as measures of clinical outcome. Samples with nonzero survival data and available status were included in the analysis.


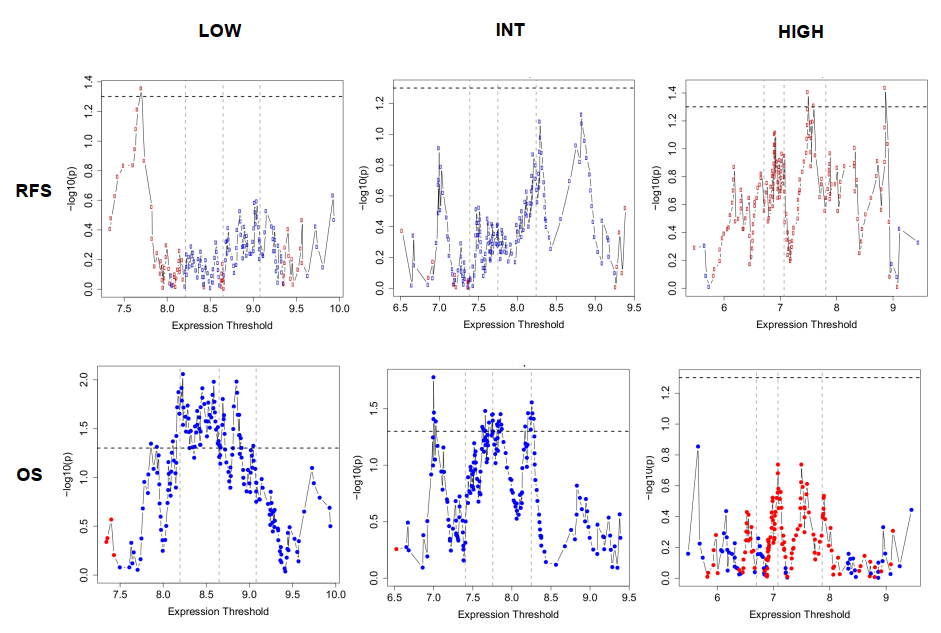


Fig. S13:

Log rank multiple cut-off graphs for AKR1B1 expression stratified by ESTIMATE scores in GSE39582. Samples divided based on stroma (upper panel) and immune (lower panel) scores. Low: Low, Int: Intermediate, High: High. OS is used as the measure of clinical outcome. Samples with nonzero survival data and available status were included in the analysis.


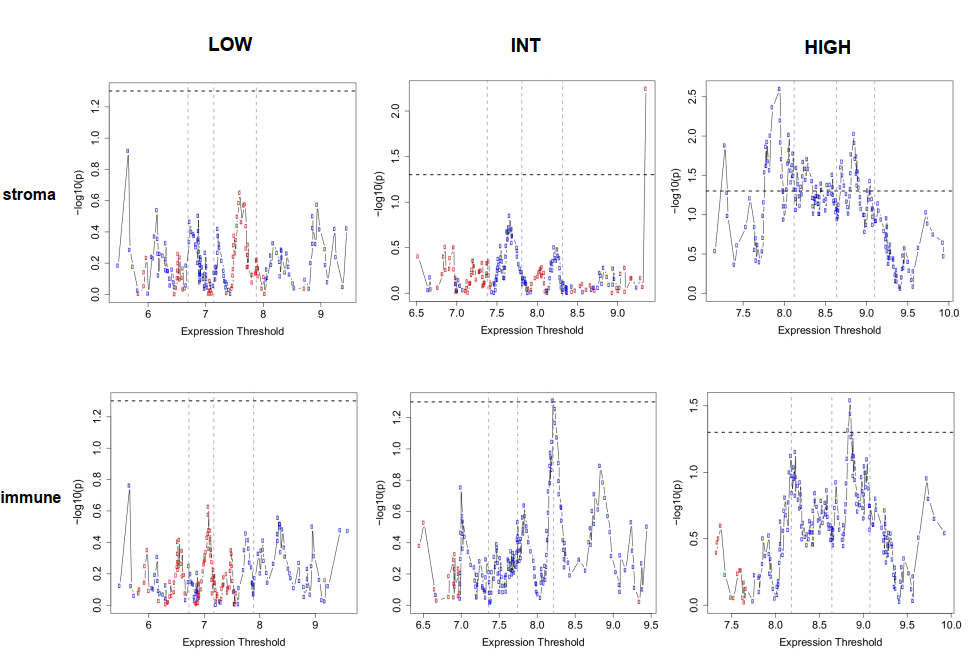


Fig. S14:

Kaplan Meier graphs of stromal B1 score. Patients were stratified based on stromal percentage of the tumor core as tumors with stromal ratio below 20 % (A) and equal to or above 20 % (B). Log rank p values are given.


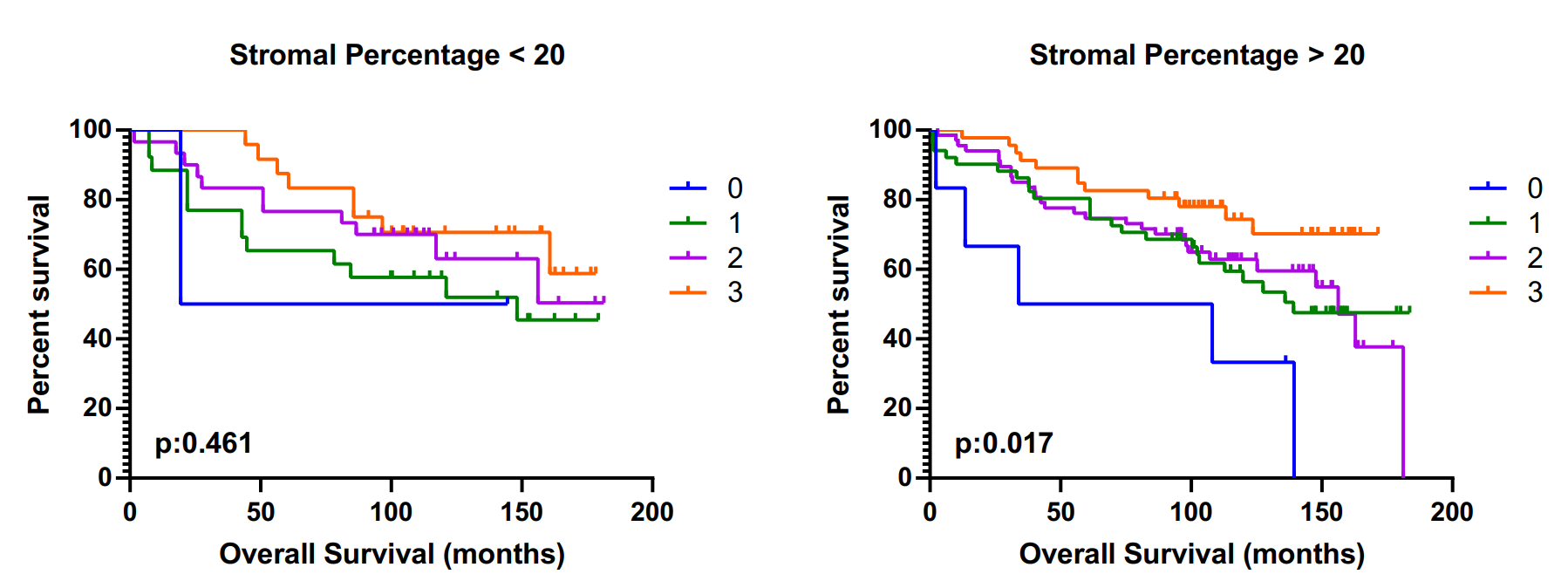

Supplement: Supplementary file 3 — Figure S8. Evaluation of prognostic relationships in the Turkish CRC cohort. Figure S9. Tumor AKR1B1 score in tumors with different clinical characteristics. Figure S10. Kaplan Meier graphs of stromal AKR1B1 score. Figure S11. Log rank multiple cutoff graphs for AKR1B1 expression. Figure S12. Log rank multiple cutoff graphs for AKR1B1 expression stratified by tumor purity in GSE39582. Figure S13. Log rank multiple cutoff graphs for AKR1B1 expression stratified by ESTIMATE scores in GSE39582. Figure S14. Kaplan Meier graphs of stromal AKR1B1 score. [file CAM4-14-e70974-s002.docx]
