# Supplementary material for: AKR1B1 Expression in the Colorectal Tumor Microenvironment Contributes Towards Its Prognostic Significance
Source: Cancer Med. 2025 May 21;14(10):e70974. doi: 10.1002/cam4.70974 (PMC12093151; doi:10.1002/cam4.70974)
Supplement: Supplementary file 4 — Appendix S1. Supplementary materials and methods. [file CAM4-14-e70974-s001.docx]

**Supplementary Materials and Methods**

**Patient characteristics**

The Turkish study cohort included formalin-fixed paraffin-embedded (FFPE) tumor tissues from 269 stage II or III CRC patients. The last follow up date or day of death was available for 264 patients. Stage, vascular and lymphatic invasion data were collected, however they were not available for all patients. The tumor location was assigned as proximal for lesions of the cecum, the ascending colon and transverse colon. Lesions from the sigmoid colon up to the splenic flexure including the splenic flexure were assigned as distal colon.

The Serbian cohort consisted of 87 FFPE tumor tissues from CRC patients with early-onset disease taken at surgery and 24 pairs of tumor and healthy mucosa samples from patients with locally advanced rectal cancer (LARC) taken at biopsy before administration of neoadjuvant therapy. Data regarding mucinous content (estimated %), grade, stage, age, gender, peritumoral and regional lymphatic invasion, perineural invasion, residual status information was available for most patients. In the early-onset disease group, there were 47 mucinous and 40 nonmucionus tumors, and the number of tumors from patients with stage I, II, III and IV disease were 7, 22, 38 and 20, respectively.

**Immunohistochemistry**

Immunohistochemistry of the Turkish cohort was carried out using citrate for antigen retrieval procedure as described previously. ^1^ Fifteen tissue microarrays (TMA) that represented 3-mm diameters of 2 cores from FFPE tissues of 264 primary colorectal cancer patients were used. TMA blocks were cut into 4-µm-thick sections. For deparaffinization, the sections were placed in the oven at 60°C, then incubated in a xylene solution for 2 x 10 min. Next, the slides were rehydrated in a series of graded alcohols (96%, 90% and 70%, respectively). Antigen retrieval was carried out in a microwave oven using citrate buffer for 10 min. Endogenous hydrogen peroxide activity was inhibited with a 7% H_2_O_2_-80% methanol solution. The slides were incubated with a primary polyclonal rabbit AKR1B1 antibody (Thermo Fisher Scientific, PA529718, Waltham, MA, USA) at 1:1600 dilution (1:800 dilution for only Fig. S7) for 60 min at room temperature. Next, a biotinylated goat anti-polyvalent secondary antibody and streptavidin peroxidase were applied for 30 min at room temperature, respectively (Thermo Scientific, Lab Vision, anti-polyvalent HRP). Signal development was carried out with 3,3′-diaminobenzidine tetrahydrochloride (DAB), followed by counterstaining with hematoxylin, and the placement of a coverslip. The intensity of AKR1B1 staining in neoplastic cells was scored as no staining (0), weak staining (1), moderate staining (2), and strong staining (3). In parallel, the percentage of stained cells was assessed. H-scores were obtained by multiplying the intensity and percentage of these staining scores, to give a number in the range of 0 to 300.

For AKR1B1 immunohistochemical staining of tumor sections from the Serbian cohort, paraffin-embedded tissue sections were deparaffinized in xylene for 2 x 5 min and further rehydrated in a series of graded ethanol solutions (100%, 96%, and 70%), 5 min each. After incubation in distilled water for 10 min, antigen retrieval was performed in a microwave (750 W) for 3×7 min (21 min total) in 0.1 M citrate buffer, pH 6.0. The sections were then washed with phosphate-buffered saline (PBS, pH 7.2) and incubated with 0.3% hydrogen peroxide in methanol for 15 min at room temperature. The tumor sections were then treated with goat blocking serum (VECTASTAIN ABC-HRP kit, PK-4001, Vector Laboratories, Newark, CA, USA; diluted according to suggested procedure) for 1 hour at room temperature. Next, the sections were incubated with the AKR1B1 antibody (1:250; Thermo Fisher Scientific, PA5-29718) was applied overnight at 4°C. For immunodetection, a VECTASTAIN® ABC-HRP kit (Rabbit IgG; Vector Laboratories, Newark, CA, USA) and the biotin-avidin system were used according to the manufacturer’s instructions. Hematoxylin was used as a counterstain, and the slides were then mounted in DPX medium (Sigma-Aldrich, St. Louis, MO, USA). For the Serbian cohort only, the mean of the scoring intensity of AKR1B1 expression was calculated by two pathologists. Staining patterns in the tumor stroma was evaluated semi-quantitatively as no staining (0), mild (1), moderate (2), and strong staining (3). No scores were assigned for cases which had staining or fixation artifacts.

Stroma and tumor percentages were evaluated for each case (Turkish and Serbian cohorts) using the hematoxylin and eosin (H&E)-stained slides as described previously. ^2,3^ Stromal percentages in the tumor center cores were evaluated per case. Evaluation was limited to a single core when the other core was unreadable (folded or sloughed off tissue, too much necrosis/hemorrhage/exudate). For the final analysis, any stromal percentage calculated on the peripheral cores were excluded to achieve uniformity. The mucin content in the mucinous samples was noted as percentages.

**Immunofluorescence staining**

FFPE tissues of normal colon and colon adenocarcinomas were sectioned into 4-µm-thick sections. For deparaffinization, the sections were incubated overnight at 60°C in xylene.^4^ Then, slides were re-hydrated in gradually in decreasing concentrations of ethyl alcohol (96%, 90%, and 70%, respectively). Heat-induced epitope retrieval was carried out in a microwave oven in 10mM EDTA buffer, pH 8.0. The sections were incubated separately with the AKR1B1 ((Thermo Fisher Scientific, PA529718, 1:3200 dilution) and the CD163 (Ab156769, Abcam, Cambridge, MA, 1:800 dilution) antibody overnight at 4°C. The sections were then incubated in the following secondary antibodies: goat antimouse IgG H&L (Alexa Fluor® 647) (ab150115, Abcam, 1:200) and goat anti-rabbit IgG H&L (Alexa Fluor® 488) (ab150077, Abcam, 1:1000) for 90 min at room temperature. The nuclei were stained with DAPI (4′,6-diamidino-2-phenylindole). Images were captured with a Leica DM2500 fluorescence microscope.

**Cell culture and macrophage differentiation**

THP-1 human monocytes were cultured in RPMI 1640 (Biological Industries, Kibbutz Beit-Haemek, Israel) medium supplemented with 10% FBS (Capricorn Scientific, Ebsdorfergrund, Germany), 2 mM L-glutamine (Biological Industries), 10 mM HEPES (AppliChem, Darmstadt, Germany), 1 mM sodium pyruvate (Biological Industries), and 4.5 g/L glucose (Gibco, Thermo Fisher) as per ATCC recommendations. The cells were grown in a humidified incubator with 5% CO_2_ at 37°C. Mycoplasma testing was regularly conducted on all cells. ^5^

THP-1 cells were differentiated into M0 macrophages using phorbol 12-myristate 13-acetate (PMA, Sigma-Aldrich, St Louis, MO, USA) and then further differentiated into M2 macrophages using standard protocols as previously described. ^6^ Briefly, viable THP-1 cells (3x10^5^ cells/ml) were treated with 5 ng/mL PMA for 24h. Next, the PMA containing medium was replaced with a fresh complete medium for 72h at 37°C. Following this, the cells were incubated with 20 ng/mL IL-4 (R&D Systems) and 20 ng/mL IL-13 (R&D Systems, Minneapolis, MN, USA) for 48h. The medium was changed to a complete medium and the cells were collected for protein or mRNA isolation after 48h of incubation.

**Western Blot**

Protein from M0 and M2 differentiated macrophages was isolated using the M-PER Mammalian Protein Extraction Reagent (Thermo Scientific) containing 1X phosphatase and protease inhibitor cocktail (Roche, Basel, Switzerland). For this, the macrophages were washed with PBS and collected by scraping in M-PER and placed into 1.5 ml centrifuge tubes. The tubes were incubated on ice for 30 min with vortexing every 10 min. Next, the samples were centrifuged at 14,000 x *g* for 10 min and the supernatant containing the total protein extract was transferred to pre-chilled centrifuge tubes and stored at -80 °C until use. The protein amount was quantified using the Bradford assay using the Coomassie Protein Assay Reagent (Thermo Scientific). The absorbance of the proteins at 595 nm was determined with a spectrophotometer (Multiskan FC Microplate Photometer, Thermo Scientific). The protein concentrations were determined with the help of a standard curve generated with bovine serum albumin.

For western blot, the proteins (30μg) were separated in 10% SDS–PAGE gels and transferred to a PVDF membrane (Roche) as described previously.^7^ The primary antibodies AKR1B1 (Invitrogen, PA5-29718), CD163 (Abcam, ab156769) and α-Tubulin (Cell Signalling Technology, 9099S) were used at 1:1000, 1:1000 and 1:5000 concentrations, respectively. The bands were visualized with the Clarity ECL Substrate (Bio-Rad) and ChemiDoc MP Imaging System (Bio-Rad). Semi-quantitative analysis of Western blot data was performed using Image Lab Software (Bio-Rad). Initially, the lanes and the corresponding bands were selected using the “Analysis Tool Box” feature of the software. To reduce any background noise, the band volumes were selected from the “Lane and Bands” feature. Subsequently, the “Quantity Tools” from the “Analysis Tool Box” were used to determine relative band intensities. A reference band, typically from the control group, was assigned a relative value of 1. The software then automatically calculated the relative intensities of all other bands based on this reference. Data from biological replicates were pooled, and statistical analysis was performed accordingly. Alpha-tubulin was used as a loading and normalization control.

**RNA isolation and Real-time PCR of tumor biopsies**

Total RNA was extracted from tumor tissues collected before neoadjuvant therapy at the First Surgical Clinic, Clinical Center of Serbia, Belgrade, Serbia using Trizol reagent (Ambion, Foster City, CA, USA). The DNAse I (Thermo Scientific) treated RNA samples (500 ng) were converted to cDNA using a RevertAid First Strand cDNA Synthesis Kit (Thermo Scientific). Fold changes in the expression of CDH1, VIM and AKR1B1 were calculated with respect to the geometric mean of two internal controls (ACTB and GAPDH) using the Pfaffl method.^8^

**RNA isolation and Real-time PCR of macrophages**

Total RNA isolation from M2 differentiated macrophages was carried out with the Monarch Total RNA Miniprep Kit (New England BioLabs, Ipswich, MA, USA) according to the manufacturer’s instructions. The RNA was quantified with BioDrop (Biochrom, Cambridge, UK). cDNA was synthesized from 1 μg of RNA sample with the RevertAid First Strand cDNA Synthesis Kit (Thermo Fisher Scientific) using random hexamer primers according to the manufacturer’s instructions. The expression of Transglutaminase 2 (TGM2) was determined by quantitative real-time PCR (qRT-PCR). The final volume of the reaction was set to 10 μl and contained 1 μl of cDNA (1:10 dilution) and 9 μl reaction mix [0.5-1 μM of Forward and Reverse primers (Table S1), 5 μl GoTaq qPCR Master Mix (Promega) and nuclease-free dH_2_O to complete the volume]. Standard curves were generated for each primer pair and β-actin was used as the internal control. The reactions were carried out in a CFX Connect Real-Time PCR Detection System (BioRad, Hercules, CA, USA) qRT-PCR equipment. Expression values of individual genes were determined using the Pfaffl method. ^8^

**Analysis of gene expression data**

Raw data for GSE17536, GSE39582, GSE39396 and GSE16385 datasets were downloaded from NCBI GEO database (https://www.ncbi.nlm.nih.gov/geo/) and RMA normalized using “affy” package. Quantile normalized gene expression data of GSE31279 dataset was downloaded from NCBI GEO and used for further analysis. For GSE16385, peripheral blood mononuclear cells (PMBCs) from two donors were analyzed. Donor 3 was not included due to inconsistencies in the replicates of the control experiments. To score colorectal (CRC) tumors for the level of immune and stromal cells in the tumor microenvironment (TME), CIBERSORT and ESTIMATE algorithms were applied to microarray data (GSE17536 and GSE39582) obtained from CRC tumors as described previously.^9,10^ The ESTIMATE algorithm was used for the scoring the level of stromal and immune contents of the tumors using microarray data.^9^ The method was applied using the “estimate” package (https://bioinformatics.mdanderson.org/estimate/rpackage.html) via R Bioconductor. Samples with low, intermediate or high stroma/immune/tumor purity scores were stratified based on ranked scores. The patients in GSE39582 were divided into approximately equal numbers in each group (Low: 188, intermediate: 189, high: 189). The CIBERSORT web tool (https://cibersort.stanford.edu/) absolute mode was used to calculate immune cell fractions for 22 different cell types for each sample. ^9^ Samples with deconvolution p value above 0.05 were eliminated to filter out the results with a poor “goodness of fit”. Markers of cancer associated fibroblasts (CAFs) were obtained from the literature. ^11,12^ For genes with multiple probesets, the probeset which showed the best linear correlation with other CAF markers was chosen. Consensus molecular subtypes (CMS) information for GSE39582 was obtained from www.synapse.org.

**Analysis of Prognostic Relationships**

Log rank multiple cut-off (LRMC) graphs were generated as described previously. ^13^ Briefly, prognostic differences were evaluated based on log-rank p values plotted on the y axis, comparing the “high” and “low” expression groups generated based on all possible cut-offs shown on the x axis. Red and blue colors indicate association of high expression with poor and good prognosis, respectively. Vertical dashed lines represent the 25 percentile, median and 75th percentile values, while the horizontal dashed line denotes p <0.05.

**Evaluation of single cell RNA-sequencing (scRNAseq) data from colorectal tumors**

scRNAseq data from colorectal tumors from the dataset GSE178318 were downloaded from NCBI GEO.^14^ Tumors of three patients who were treatment naive were included in the analysis. Data was processed via "Seurat" package in R Bioconductor. ^15^ Quality filtering was carried out as follows: cells with expressed features < 500 and > 6000, cells with mitochondrial gene expression >15% were filtered out. Doublets were detected and removed using the "DoubletFinder" package. ^16^ The UMAP method was used for dimension reduction.

The filtered data of GSE178341 was downloaded and used for visualization. ^17^ Briefly, the filtering criteria were as follows: cell barcodes were excluded if they satisfied any one of the following criteria: 1. Fewer than 200 genes; 2. Fewer than 1,000 reads; 3. Fewer than 500 UMIs; 4. More than 50% of UMIs mapping to the mitochondrial genome; 5. Non-empty droplet with false discovery rate (FDR) less than 0.1; 6. Over 5% of reads estimated to come from swapped barcodes/chimeric reads. This dataset included tumors from 28 MMRp (mismatch repair proficient) and 34 MMRd (mismatch repair deficient) patients which were used in this study. Single cell RNA seq data from GSE146771 was analyzed with a previously published web portal <http://crcleukocyte.cancer-pku.cn/>. ^18^

**Statistical Analysis**

The medians of two groups were compared by Mann Whitney U test. The comparison of AKR1B1 expression between CMS groups was carried out with One-way ANOVA followed by Tukey’s multiple comparison test. Wilcoxon matched-pairs signed rank test was conducted for the comparison of expression in paired tumor and normal tissues and Kaplan Meier graphs were generated using Graphpad Prism 9 (San Diego, CA, USA). For the Turkish cohort, overall survival (OS) in months was calculated as “(days between the last follow-up date/date of death and the date of diagnosis)/30”. Recurrence-free survival (RFS) in months was calculated as “(days between the date of recurrence and the date of diagnosis)/30”. Pearson correlation analysis was carried out using Microsoft Excel 2016 using “PEARSON” formula and Spearman correlation analyses were conducted using Graphpad Prism 9.

**References**

1 Akyol A, Güner G, Özşeker HS, Işık A, Atcı Ö, Uzun S *et al.* An immunohistochemical approach to detect oncogenic CTNNB1 mutations in primary neoplastic tissues. *Laboratory Investigation* 2019; 99(1):128-137. doi:10.1038/s41374-018-0121-9.

2 van Pelt GW, Kjær-Frifeldt S, van Krieken JHJM, Al Dieri R, Morreau H, Tollenaar RAEM *et al.* Scoring the tumor-stroma ratio in colon cancer: procedure and recommendations. *Virchows Archiv* 2018; 473(4):405-412. doi:10.1007/s00428-018-2408-z.

3 Smit MA, van Pelt GW, Terpstra V, Putter H, Tollenaar RAEM, Mesker WE *et al.* Tumour-stroma ratio outperforms tumour budding as biomarker in colon cancer: a cohort study. *Int J Colorectal Dis* 2021; 36(12):2729-2737. doi:10.1007/s00384-021-04023-4.

4 Uzun S, Isik A, Katipoglu K, Guner G, Akyol A. Characterization of the Subcellular Distribution of Phospho-β-catenin in Colorectal Cancer. *In Vivo (Brooklyn)* 2023; 37(4):1576-1583. doi:10.21873/invivo.13242.

5 Young L, Sung J, Masters JR. Detection of mycoplasma in cell cultures. *Nat Protoc* 2010; 5(5):929-34. doi:10.1038/nprot.2010.43.

6 Baxter EW, Graham AE, Re NA, Carr IM, Robinson JI, Mackie SL *et al.* Standardized protocols for differentiation of THP-1 cells to macrophages with distinct M(IFNγ+LPS), M(IL-4) and M(IL-10) phenotypes. *J Immunol Methods* 2020; 478:112721. doi:10.1016/j.jim.2019.112721.

7 Demirkol Canlı S, Seza EG, Sheraj I, Gömçeli I, Turhan N, Carberry S *et al.* Evaluation of an aldo-keto reductase gene signature with prognostic significance in colon cancer via activation of epithelial to mesenchymal transition and the p70S6K pathway. *Carcinogenesis* 2020; 41(9):1219-1228. doi:10.1093/carcin/bgaa072.

8 Pfaffl MW. A new mathematical model for relative quantification in real-time RT-PCR. *Nucleic Acids Res* 2001; 29(9):e45. doi: 10.1093/nar/29.9.e45.

9 Yoshihara K, Shahmoradgoli M, Martínez E, Vegesna R, Kim H, Torres-Garcia W *et al.* Inferring tumour purity and stromal and immune cell admixture from expression data. *Nat Commun* 2013; 4:2612. doi:10.1038/ncomms3612.

10 Newman AM, Liu CL, Green MR, Gentles AJ, Feng W, Xu Y *et al.* Robust enumeration of cell subsets from tissue expression profiles. *Nat Methods* 2015; 12(5):453-7. doi:10.1038/nmeth.3337.

11 Son GM, Kwon MS, Shin DH, Shin N, Ryu D, Kang CD. Comparisons of cancer-associated fibroblasts in the intratumoral stroma and invasive front in colorectal cancer. *Medicine (United States)* 2019; 98(18):e15164. doi:10.1097/MD.0000000000015164.

12 Liu T, Han C, Wang S, Fang P, Ma Z, Xu L *et al.* Cancer-associated fibroblasts: An emerging target of anti-cancer immunotherapy. J Hematol Oncol. 2019; 12(1):86. doi:10.1186/s13045-019-0770-1.

13 Demirkol S, Gomceli I, Isbilen M, Dayanc BE, Tez M, Bostanci EB *et al.* A combined ULBP2 and SEMA5A expression signature as a prognostic and predictive biomarker for colon cancer. *J Cancer* 2017; 8(7):1113-1122. doi:10.7150/jca.17872.

14 Che LH, Liu JW, Huo JP, Luo R, Xu RM, He C *et al.* A single-cell atlas of liver metastases of colorectal cancer reveals reprogramming of the tumor microenvironment in response to preoperative chemotherapy. *Cell Discov* 2021; 7(1):80. doi:10.1038/s41421-021-00312-y.

15 Satija R, Farrell JA, Gennert D, Schier AF, Regev A. Spatial reconstruction of single-cell gene expression data. *Nat Biotechnol* 2015; 33(5):495-502. doi:10.1038/nbt.3192.

16 McGinnis CS, Murrow LM, Gartner ZJ. DoubletFinder: Doublet Detection in Single-Cell RNA Sequencing Data Using Artificial Nearest Neighbors. *Cell Syst* 2019; 8(4):329-337.e4. doi:10.1016/j.cels.2019.03.003.

17 Pelka K, Hofree M, Chen JH, Sarkizova S, Pirl JD, Jorgji V *et al.* Spatially organized multicellular immune hubs in human colorectal cancer. *Cell* 2021; 184(18):4734-4752.e20. doi:10.1016/j.cell.2021.08.003.

18 Zhang L, Li Z, Skrzypczynska KM, Fang Q, Zhang W, O’Brien SA *et al.* Single-Cell Analyses Inform Mechanisms of Myeloid-Targeted Therapies in Colon Cancer. *Cell* 2020; 181(2):442-459.e29. doi:10.1016/j.cell.2020.03.048.
