# Supplementary material for: AKR1B1 Expression in the Colorectal Tumor Microenvironment Contributes Towards Its Prognostic Significance
Source: Cancer Med. 2025 May 21;14(10):e70974. doi: 10.1002/cam4.70974 (PMC12093151; doi:10.1002/cam4.70974)
Supplement: Supplementary file 5 — Table S1. Primer pairs used in the study. [file CAM4-14-e70974-s004.docx]

Supplementary Table 1: Primer pairs used in the study

| **Target Gene** | **Forward Primer (5’→3’)** | **Reverse Primer (5’→3’)** | **Tm (°C)** |
| --- | --- | --- | --- |
| AKR1B1 NM_001628.4 | AAGCCGTCTCCTGCTCA | TTGCTGACGATGAAGAGC | 55 |
| TGM2  NM_001323316.2, NM_001323317.2, NM_001323318.2, NM_004613.4 | GCAGTGACTTTGACGTCTTTGCCC | GTAGCTGTTGATAACTGGCTCCACG | 61 |
| ACTB  NM_001101.5 | CAGCCATGTACGTTGCTATCCAGG | AGGTCCAGACGCAGGATGGCATG | 55-61 |
| GAPDH  NM_001357943.2, NM_001256799.3, NM_001289745.3, NM_001289746.2, NM_002046.7 | CGACCACTTTGTCAAGCTCA | CCCCTCTTCAAGGGGTCTAC | 58 |
